# A chromosome‐scale genome assembly of *Hordeum erectifolium* : genomic, transcriptomic and anatomical adaptations to drought in a wild barley relative

**DOI:** 10.1111/nph.71091

**Published:** 2026-03-24

**Authors:** Einar Baldvin Haraldsson, Michael Anokye, Thea Rütjes, Helena Toegelová, Zuzana Tulpová, Hana Šimková, Jia‐Wu Feng, Martin Mascher, Maria von Korff

**Affiliations:** ^1^ Institute of Plant Genetics Heinrich‐Heine‐University Düsseldorf 40225 Düsseldorf Germany; ^2^ Institute of Experimental Botany of the Czech Academy of Sciences 78371 Olomouc Czech Republic; ^3^ Leibniz Institute of Plant Genetics and Crop Plant Research (IPK) Gatersleben 06466 Seeland Germany; ^4^ German Centre for Integrative Biodiversity Research (iDiv) Halle‐Jena‐Leipzig 04103 Leipzig Germany; ^5^ Cluster of Excellence on Plant Sciences (CEPLAS) 40225 Düsseldorf Germany

**Keywords:** comparative transcriptomics, drought, drought adaptation, evolution, genome assembly, *Hordeum erectifolium*, *Hordeum vulgare*, Poaceae

## Abstract

- Wild crop relatives are valuable genetic resources for improving stress adaptation in cultivated species, but their effective use depends on high‐quality reference genomes integrated with phenotypic and molecular datasets. *Hordeum erectifolium*, a wild relative of barley (*H. vulgare*), is adapted to intermittent and prolonged drought and saline soils, making it an excellent species for stress‐adaptation research.
- We assembled a chromosome‐scale, annotated reference genome of *H. erectifolium* comprising 3.85 Gbp, and identified 71 475 genes supported by a tissue‐specific gene expression atlas. Comparative morphological, physiological and transcriptomic analyses under water limitation were conducted with *H. erectifolium*, and cultivated and wild barley.
- *Hordeum erectifolium* displayed a greater density of leaf veins and sclerenchyma cells, alongside rapid leaf rolling upon dehydration. Genomic comparisons revealed structural variations, independent transposon‐driven evolution and copy number expansions of desiccation‐responsive gene families relative to barley. The transcriptional responses of *H. erectifolium* and barley to water limitation suggested contrasting drought‐adaptation strategies: metabolic downregulation and survival prioritization in *H. erectifolium* vs maintenance of metabolic activity and competitiveness in barley.
- Our data suggest that *H. erectifolium* is genetically primed for survival under drought through anatomical adaptations, gene family expansion, efficient shutdown of growth‐related metabolism and rapid recovery upon rehydration.

## Introduction

To ensure food security in the face of a changing climate, it is essential to develop and implement innovative strategies that enhance crop resilience to extreme weather events, diversify food sources and optimize resource use. Crop wild relatives (CWR), the close wild relatives of domesticated crop species, harbor novel traits and alleles that serve as a valuable resource for breeding stress‐resilient crops and for advancing research into the genetic and genomic basis of plant adaptation (Feuillet *et al*., 2008; Bohra *et al*., 2022).

CWRs are categorized into three gene pools. The primary gene pool comprises the cultivated crop and its closest wild relatives that can cross easily and produce fertile offspring. Secondary relatives can be crossed with some difficulty and reduced fertility, while tertiary relatives require advanced methods such as genetic engineering or wide hybridization to overcome strong reproductive barriers (Harlan & de Wet, 1971; Baker *et al*., 2020; Kashyap *et al*., 2022). For over a century, CWRs have been used for crop improvement, in particular as a resource for disease and pest resistance genes, but also to improve resistance to abiotic stresses (Hajjar & Hodgkin, 2007; Renzi *et al*., 2022). The introgression of stress escape and avoidance strategies into elite germplasm has often been achieved by introducing exotic alleles that alter phenology, the timing of germination and reproductive development (Duc *et al*., 2015; Farooq *et al*., 2025). More recently, CWRs have been used to introduce perennial growth, a complex trait that extends survival and allows for reproduction over multiple seasons, into annual crops (Gruner & Miedaner, 2021; Zhang *et al*., 2023). Perennial crops promise to reduce labor costs, decrease input requirements, mitigate soil erosion and enhance soil health, thereby supporting more sustainable agriculture (Zhang *et al*., 2023). However, the introgression of novel traits such as perenniality, disease and abiotic stress resistance from CWR is hampered by hybridization barriers, low fertility and time and labor costs for generating advanced lines (Wendler *et al*., 2015). The availability of reference genomes and genetic tools for gene identification and transfer now promises to deliver novel, efficient approaches for using CWR in research and breeding (Brozynska *et al*., 2016; Gao *et al*., 2024).

The annual crop barley (*Hordeum vulgare* ssp. *vulgare*) was domesticated from the wild barley ancestor *H. vulgare* ssp. *spontaneum* in the Fertile Crescent (Badr *et al*., 2000; Pankin *et al*., 2018). Wild and cultivated barley belong to the primary gene pool and are characterized by natural continuous gene flow in the Fertile Crescent, where wild and cultivated barley co‐occur (Pankin *et al*., 2018). Wild barley has been successfully used to introgress novel alleles for disease resistance, abiotic stress tolerance and flowering time into elite barley (Matus *et al*., 2003; von Korff *et al*., 2004; Hernandez *et al*., 2020). In addition, *H. bulbosum* from the secondary CWR gene pool was a source of resistance genes to powdery mildew, leaf rust, barley mild and yellow mosaic virus for breeding elite barley (Walther *et al*., 2000; Vincent *et al*., 2012). However, CWRs from the tertiary gene pool within the *Hordeum* genus have not yet been used for barley improvement, not only because of hybridization barriers but also because of a lack of genetic and genomic resources. The *Hordeum* genus, comprising *c*. 33 annual and perennial species, originated in the Mediterranean some 12 Ma and has since migrated globally, with 16 species in South America, which have evolved in the last 1.5 Ma (Blattner, 2018). The South American clade of the *Hordeum* genus contains closely related species adapted to different ecological niches and with different life history strategies, making them an interesting resource for trait diversification and research into the physiological and genetic underpinnings of adaptation (von Bothmer & Komatsuda, 2010). Among the South American clade, *H. erectifolium* is one of the younger species, with the last common ancestor of *H. erectifolium* and its closest sister species, *H. stenostachys*, dating back to *c*. 1.3 Ma, whereas the split of the branches separating *H. erectifolium* and *H. vulgare* occurred *c*. 9.2 Ma (Brassac & Blattner, 2015). *H. erectifolium* is endemic to the southernmost part of the semiarid Pampas, Argentina, where it grows among halophytes near a salt lake (von Bothmer *et al*., 1985). This region is impacted by frequent irregular periods of drought that can extend to up to a year (von Bothmer *et al*., 1985; Cai *et al*., 2020; WMO, 2020; Sgroi *et al*., 2021). Due to its small distribution area, it is considered a near‐threatened species (IUCN, 2024) and is listed as a high priority for *ex situ* conservation within the *Hordeum* genus; only a single accession of *H. erectifolium* is available in gene banks (Vincent *et al*., 2012). Being a perennial species, *H. erectifolium* has to persist through long and unpredictable periods of drought and saline soil. It has thus evolved unique stress avoidance traits, such as very glaucous erect basal leaves and pubescence on the leaf abaxial side, a thick wax coating and large suberized silica cells (von Bothmer *et al*., 1985, 1995). These unique adaptations make *H. erectifolium* an interesting genetic resource to explore the physiological and genetic underpinnings of stress adaptation compared with the closely related cultivated crop barley.

We present a chromosome‐scale, annotated reference genome of *H. erectifolium*, together with a comprehensive tissue‐specific gene expression atlas. By comparing genomic, transcriptomic and morphological responses to water limitation with those of cultivated barley, we highlight stress‐adaptive strategies employed by *H. erectifolium*. Our findings demonstrate the value of this perennial close relative of barley for dissecting the morphological, physiological and genetic bases of stress adaptation.

## Materials and Methods

### Plant material and growth conditions

For all experiments, we used single descent propagated seeds of *Hordeum erectifolium* Bothmer, N.Jacobsen & R.B.Jørg. acc. NGB6816 (Nordic Genetic Resource Center, Sweden), and *Hordeum vulgare* var. *spontaneum* (K.Koch) Körn. acc. B1K‐04‐12 from the Barley1K collection (Hübner *et al*., 2009), *Hordeum vulgare* var. *spontaneum* (K.Koch) Körn. acc. HID‐4 (Liller *et al*., 2017), and *H. vulgare* L. cv Morex. *H. erectifolium* and Morex were used in all experiments, but B1K‐04‐12 was used for leaf anatomical phenotyping, and HID‐4 for specific leaf area (SLA) and elemental carbon and nitrogen measurements. Unless specified otherwise, plants were consistently grown under the following conditions. Seeds were sown in a mixture of 93% (v/v) Einheitserde ED73 (Einheitserdewerke Werkverband e.V., Sinntal‐Altengronau, Germany), 6.6% (v/v) sand and 0.4% (v/v) Osmocote exact standard 3–4 M (ICL Deutschland Vertriebs GmbH, Nordhorn, Niedersachsen, Germany). Stratified at 4°C before being placed in a growth chamber with long‐day conditions (16 h : 8 h, 20°C : 16°C, light : dark, 60% relative humidity) for germination. Ten days after germination, they were vernalized for 8 wk at 4°C under short‐day conditions (8 h : 16 h, 4°C : 4°C, light : dark), before being transferred back to long‐day conditions. Plants used for SLA and elemental carbon and nitrogen measurements were cultivated as described previously. After vernalization, they were repotted to 7.5‐l pots and grown further in a common garden at the Botanical Garden of the Heinrich‐Heine‐Universität Düsseldorf (HHU), data collected were from three summer seasons of 2021–2023.

### Phenotyping

To visualize the leaf anatomy, we used toluidine blue to stain the lignin of transversely cut leaves. We collected the flag leaf and the leaf below it, and 1‐cm sections at the midpoint of each leaf were thinly sliced and fixed for staining with toluidine blue for microscope image capture. The SLA was measured by scanning the area (cm^2^) of a main culm flag leaf with Petiole (Petiole LTD, USA), an application on a mobile device. The dry weight (mg) was measured after drying at 65°C, and SLA was calculated as SLA = mm^2^ mg^−1^. We measured elemental carbon and nitrogen by pooling the flag leaves from the first three reproductive tillers per plant at the grain filling stage. They were dried at 65°C before being pulverized to a fine powder, and *c*. 2 mg was accurately measured. The samples were provided to the CEPLAS Metabolomics and Metabolism Laboratory (CMML, Heinrich‐Heine University Düsseldorf, Germany) for measurements of elemental carbon and nitrogen.

### Generation of an annotated chromosome‐scale genome assembly

A single plant of *H. erectifolium* acc. NGB6816, grown under control conditions, was used for extracting high‐molecular‐weight (HMW) DNA. HMW DNA was extracted from young leaf material and sequenced on 27 Oxford Nanopore Technology (ONT) flow cells (Supporting Information Table S1) and with 10× Genomics Linked‐Reads sequencing for assembly polishing and the first scaffolding. Bionano optical genome mapping was performed using material of the same plant that also provided the seeds used to generate seedlings for Hi‐C chromosome conformation capture sequencing. We processed the 10× Genomics Linked‐Reads data with Long Ranger (10× Genomics, 2024), generating paired‐end Illumina (PE150) short‐reads data with Linked‐Reads information in headers (short‐reads). We performed initial genome *k*‐mer‐based characterization (size, ploidy level and heterozygosity) by analyzing the short‐reads data using Jellyfish and findGSE (Marçais & Kingsford, 2011; Sun *et al*., 2018).

The ONT sequencing was basecalled with Guppy (Oxford Nanopore Technologies, 2024) using a quality threshold of ≥ Q7 and subsequently trimmed sequencing adapters with Porechop (Wick *et al*., 2017), before assembling the ONT long‐reads with Flye (Kolmogorov *et al*., 2019). The assembly was iteratively polished; first, we used minimap2 to map the ONT long‐reads to the assembly before each polishing step, first with Racon and then with Medaka (Vaser *et al*., 2017; Li, 2018; Oxford Nanopore Technologies, 2024). Thereafter, we mapped the short‐reads with BWA‐MEM2 to the long‐read polished assembly and performed two rounds of polishing with Hapo‐G and repeated the mapping between rounds (Vasimuddin *et al*., 2019; Aury & Istace, 2021). The polished assembly was progressively scaffolded from contigs to pseudomolecules. Initially, Tigmint was used with the 10× Genomics Linked‐Reads short‐read data (Jackman *et al*., 2018), followed by hybrid scaffolding using Bionano optical genome mapping (Šimková *et al*., 2023). Finally, the assembly was organized into pseudomolecules using the TRITEX pipeline with Hi‐C chromosome conformation capture sequencing (Monat *et al*., 2019). Assembly quality, completeness and metrics were assessed at each step using complementary tools: Merqury for *k*‐mer–based evaluation of assembly accuracy and *k*‐mer completeness, BUSCO for gene space completeness and QUAST for standard assembly statistics (Mikheenko *et al*., 2018; Rhie *et al*., 2020; Manni *et al*., 2021). A unique genome assembly identifier, lpHorErec1.1, was registered at ToLID – Tree of Life Identifiers (The Tree of Life Programme, 2025).

We conducted a comprehensive tissue‐time specific transcriptome profiling of *H. erectifolium*. All mature RNA plant tissue samples and seeds used for seedlings and germinating seed samples were collected from the same plant as was previously harvested for HMW DNA. We selected 12 tissues, 10 of which were sampled at two time points during the day, in the morning (MOR, ZT 1–3) and evening (EVE, ZT 13–15), a total of 22 samples. The vegetative tissues contained 3‐d‐old germinating seeds (GS3), whole roots (RO8, 8 d post germination) and whole shoots (LS8, 8 d post germination). At anthesis, the third nodes (NOD), fourth internodes (INT) and flag leaves (FLF) were collected. The six reproductive samples consisted of developing spikes at the spikelet induction phase (ESP, W3.0–4.5), at floral development (MSP, W5.0–6.5) and during rapid spike and floret growth (LSP, W7.0–8.0), anthers (ANT) and ovules (OVU) were individually dissected at flowering (W10) and caryopses (CAR, 10 d post anthesis) (Waddington *et al*., 1983). RNA was extracted, and each tissue was barcoded and sequenced with PacBio IsoSeq (Pacific Biosciences, USA).

For gene annotation, we first mapped the 22 PacBio IsoSeq samples to the assembled genome and processed them with the isoseq3 pipeline (PacBio, 2024). We predicted the open reading frames (ORF) for each isoform using TransDecoder (Haas, 2023), and additional *ab initio* structural gene annotation was performed with Helixer (Holst *et al*., 2025). The IsoSeq‐Transdecoder and Helixer results were merged for a final gene annotation, and functionally annotated with InterProScan and Mercator4 (Jones *et al*., 2014; Bolger *et al*., 2021). We further predicted the presence of lncRNA in the IsoSeq data. Protein coding gene predictions were classified into high‐(HC) or low‐confidence (LC) categories by aligning them to three manually curated databases; UniMag (reviewed Magnoliopsida), and UniPoa (Poaceae, reviewed and unreviewed) (The UniProt Consortium *et al*., 2023), and PTREP (TREP release 19), a database of hypothetical transposable element (TE) proteins (Schlagenhauf & Wicker, 2016). We classified a gene as HC when it had a best hit in UniMag, or in UniPoa, but not in PTREP (hypothetical TE proteins); otherwise, it was classified as LC. Transcript abundance in 22 tissue‐ and time‐specific samples was quantified as normalized transcripts per million using IsoQuant (Prjibelski *et al*., 2023), and the samples were clustered using principal component analysis (PCA). We visualized the top 20 contributing genes, separating the 22 tissues along the principal components 1 (PC1) and PC2.

For genomic and genetic comparisons between *H. erectifolium* acc. NGB6816 and barley, we used the barley reference genome MorexV3, and the genome of a wild barley *Hv*. *spontaneum* acc. B1K‐04‐12 (Jayakodi *et al*., 2020; Mascher *et al*., 2021). We annotated TE and repetitive regions of the *H. erectifolium* and barley genomes with EDTA and further classified long terminal repeat (LTR) superfamilies with TEsorter (Ou *et al*., 2019; Zhang *et al*., 2022). The telomeric ends were identified by aligning the telomeric sequence TTTAGGG^8^ and centromeres using the *CRM* coding domains, extracted with TEsorter from the EDTA annotation of LTR retrotransposon (Ou *et al*., 2019; Zhang *et al*., 2022). The sequences were aligned with BLASTN to the three genomes (Camacho *et al*., 2009). We identified the centromere midpoints from characteristic alignment density patterns using hdrcde (Hyndman *et al*., 2023). We calculated the chromosomal synteny among the genomes by mapping them against one another with minimap2 (Li, 2018), and structural variations were calculated with Synteny and Rearrangement Identifier (SyRI) before visualization with plotsr (Goel *et al*., 2019; Goel & Schneeberger, 2022). A more detailed description is found in Methods S1.

### Gene family evolution analysis with Orthofinder and CAFE5

A study of unique and shared genes and gene families was made between *H. erectifolium* and eight additional species, including the two barley genotypes. The proteomes of seven species were retrieved from the JGI Phytozome database: *Arabidopsis thaliana* (L.) Heynh., *Sorghum bicolor* (L.) Moench, *Zea mays* L., *Oryza sativa* L., *Brachypodium distachyon* (L.) P.Beauv., *Triticum aestivum* L. cv Chinese Spring and *Thinopyrum intermedium* (Host) Barkworth & D.R.Dewey (Goodstein *et al*., 2012). We assigned the proteomes to Hierarchical Orthologous Groups (HOG) with Orthofinder, and gene family evolution analyzed with CAFE5 (Emms & Kelly, 2019; Mendes *et al*., 2021), with a species separation time of 59 Myr (providing a separation time between *S. bicolor* and *H. erectifolium* of 59 Ma) retrieved from timetree5 (Kumar *et al*., 2022).

### Cross‐species drydown experimental setup and differential gene expression analysis

We conducted a drydown and recovery experiment to study the cross‐species transcriptomic and physiological responses to a reduction in relative leaf water content. One seed was sown per 7 × 7 × 8 cm pot with 150 ± 1 g of soil, and plants were germinated and further cultivated under 12 h : 12 h, 20°C : 16°C, light : dark, 60% relative humidity. *H. erectifolium* plants were grown for 8 wk, and Morex plants for 2 wk to ensure comparable biomass at the start of the treatment. Soil moisture was adjusted to 50% field capacity (FC), control FC and water was withheld for 6 d before rewatering to control FC. Four replicate samples of the second leaves from the main tillers were collected for transcriptomic analyses at 4 d of treatment (DOT) at ZT 8, on DOT 2, 5, 6 and 7, 24 h after rewatering. Leaf relative water content (RWC) was measured at each DOT. Total RNA was extracted, and the transcriptome was Illumina PE150 sequenced.

The RNAseq reads were mapped to their respective genomes with STAR, and quantification was done using featureCounts (Dobin *et al*., 2013; Liao *et al*., 2014). The quantified reads were processed and normalized with edgeR, and differentially expressed genes (DEG) were analyzed at individual time points with edgeR and maSigPro was used for time‐course and co‐expression analysis of DEGs in response to treatment over time (Nueda *et al*., 2014; Chen *et al*., 2025). Gene Ontology (GO) functional enrichment was performed using ClusterProfiler (Xu *et al*., 2024). Single copy orthologs between *H. erectifolium* and Morex were retrieved from HOGs and were used for direct DEG comparison between species.

### Data analysis

Data wrangling, statistics and visualization were performed in R (v.4.4.3) (R Core Team, 2024), utilizing tidyverse (v.2.0.0), and statistics with agricole (v.1.5) (Wickham *et al*., 2019; de Mendiburu, 2023).

## Results

### Leaf morphology of *H. erectifolium*, cultivated and wild barley

**Fig. 1:**
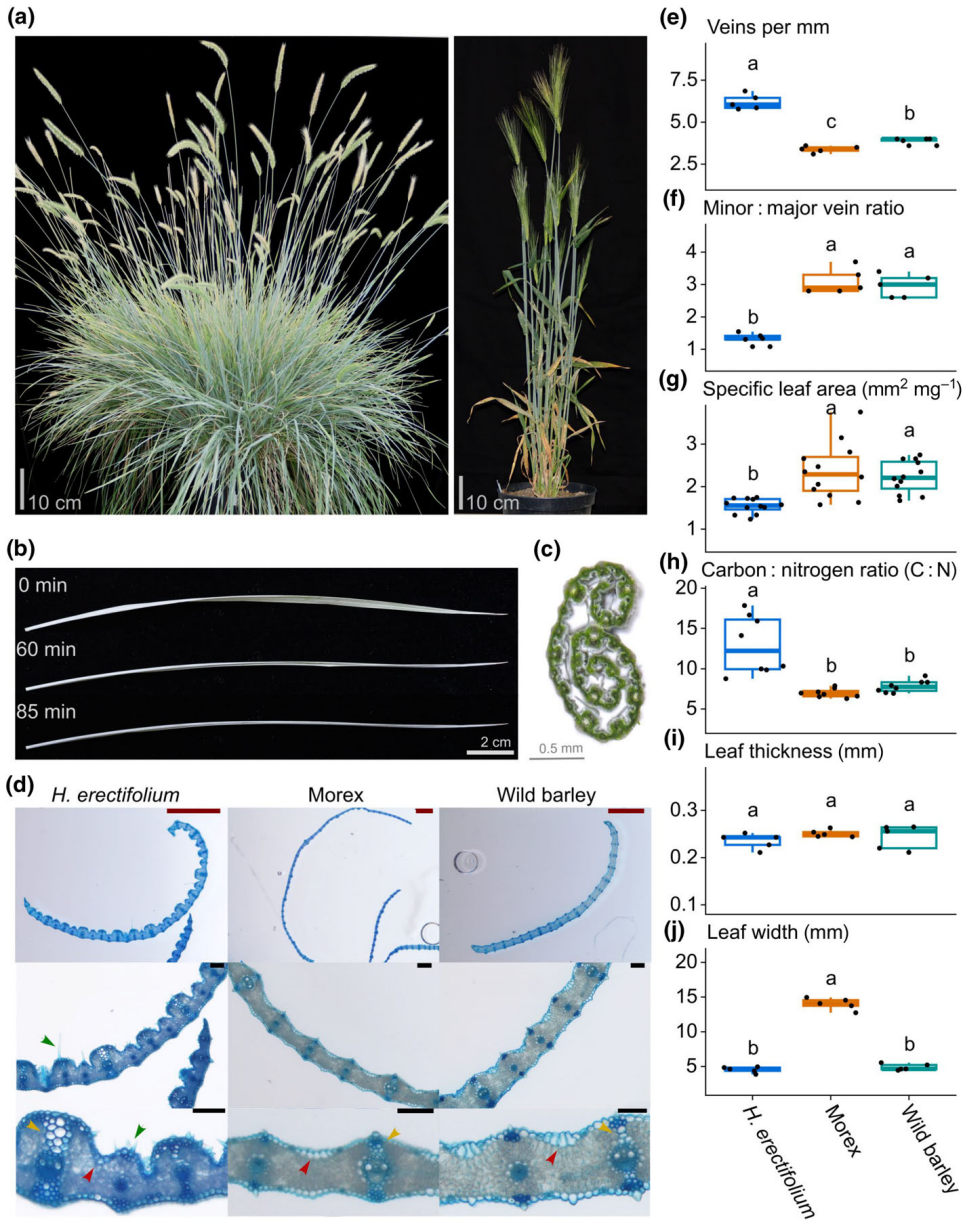
Leaf morphology of *Hordeum erectifolium*, cultivated (Morex) and wild barley. (a) A representative image of *H. erectifolium* acc. NGB 6816 (left) and an example of *H. vulgare* cv Morex (right). (b) Abscised leaf of *H. erectifolium*, folding inward toward the adaxial side of the midrib; the leaf appears white due to a thick surface wax layer. (c) Transverse cut of an *H. erectifolium* leaf, rolled toward the adaxial side of the midrib. (d) Transverse cuts of *H. erectifolium*, Morex and wild barley; lignin of fixated tissue was stained with toluidine blue. Red arrows point at bulliform cells in Morex and wild barley, and the expected location in *H. erectifolium*. Trichomes are indicated by green arrows in *H. erectifolium*. Yellow arrows point to the adaxial bundle sheath extensions (BSE) on a major vein; the BSE is also on the abaxial side of the vein. Bars for 1000 μm are indicated in red and 100 μm in black. Quantitative leaf characteristics of the flag leaf (FL) in *H. erectifolium*, Morex, and wild barley: (e) number of veins per mm, *n* = 5, (f) minor to major vein ratio, *n* = 5, (g) specific leaf area, mm^2^ mg^−1^, *n* = 8, (h) ratio of elemental carbon and nitrogen, *n* = 8. (i, j) FL thickness and width, mm, *n* = 5. Boxplots (e–j) show the distribution of values (black dots); center line shows the median, lower and upper hinges denote the first and third quartile, respectively, and the upper and lower whiskers extend no further than 1.5 times the inter‐quartile range of their respective hinge. Different letters indicate significantly different groups, ANOVA with *post hoc* Tukey's HSD, *P* < 0.05.

*Hordeum erectifolium* leaves were erect and had a glaucous appearance (Fig. 1a,b). We observed that upon reduced water availability, *H. erectifolium* rolled its leaves, whereas wild and cultivated barley maintained flat leaves (Fig. 1b,c). Leaf rolling is typically supported by specific leaf anatomies, such as reduced or a lack of bulliform cells, which provide structural support (Redmann, 1985). We therefore compared the leaf anatomy of the flag leaf and the first leaf below the flag leaf of *H. erectifolium*, cultivated (Morex) and wild barley. Here, we present the results for the flag leaf, which were confirmed in the first leaf below (Fig. S1). The flag leaf of *H. erectifolium* was characterized by a prominent ribbed leaf structure with short and long trichomes on the adaxial side, with no discernible bulliform cells. We also observed that as the flag leaf of *H. erectifolium* develops and extends, the abaxial side generally faces upward. The white appearance of the flag leaf indicated a layer of cuticular wax (Fig. 1a,b). By contrast, cultivated and wild barley both had flat leaf surfaces, much shorter trichomes, and pronounced bulliform cells (Fig. 1d). We quantified the traverse vein density, the minor: major vein ratio, leaf thickness and width, as well as SLA and elemental carbon: nitrogen ratio. Major veins are distinguished from minor veins by their additional bundle sheath extensions (BSE) (Perico *et al*., 2022). The average number of veins per mm was 6.2 vs 3.4 and 3.9 veins mm^−1^, in *H. erectifolium* vs cultivated and wild barley genotypes, respectively (Figs 1e, S1). The minor : major vein ratio had significantly shifted in *H. erectifolium* compared to cultivated and wild barley, from 1.3 vs 3.0 and 3.1, respectively. While cultivated and wild barley had three minor veins flanked by major veins, the central minor vein had become a major vein in *H. erectifolium* (Figs 1d,f, S1). We noted that *H. erectifolium* had overall more extensive sclerenchyma cells within its BSE on the adaxial side of the veins compared with barley. Sclerenchyma cells are associated with increased photosynthetic activity, water transport and more rapid stomatal closure in addition to structural support (Buckley *et al*., 2011). We found that SLA was significantly lower and carbon: nitrogen (C : N) ratios were significantly higher in *H. erectifolium* than in cultivated and wild barley; SLA with 1.5 mm^2^ mg^−1^ vs 2.4 mm mg^−1^ and 2.2 mm mg^−1^, and C : N ratio of 13.0 vs 7.0 and 7.8, in *H. erectifolium* and cultivated and wild barley, respectively (Fig. 1g,h). Low SLA and high C : N ratio indicate a slow investment in leaf growth and the photosynthetic apparatus, and thus a conservative growth strategy with improved resource use efficiency (Poorter *et al*., 2009; Onoda *et al*., 2017). Leaf thickness and width were overall not different between species; however, the leaf width of Morex was significantly greater than that of wild barley and *H. erectifolium* (Figs 1i,j, S1).

In short, *H. erectifolium* exhibited significantly higher leaf venation and a greater number of major veins – likely due to the conversion of minor veins into major ones – as well as a higher abundance of sclerenchyma cells on both the abaxial and adaxial sides of the vascular bundles, indicating greater photosynthetic tissue compartmentalization and water distribution efficiency.

### A complete, annotated *H. erectifolium* reference genome

Motivated by the distinct leaf morphology of *H. erectifolium* and reports describing its region of origin as drought‐prone and saline (von Bothmer *et al*., 1985), we investigated its genomic and transcriptomic differences relative to the barley cultivar Morex. *H. erectifolium*, like barley, has a large diploid (2*n* = 14) genome, reported with a haploid size of 2*n* = 9.49 ± 0.05 pg, *c*. 4.6 Gbp by flow cytometry, and the genome size of *H*. *vulgare* at 5.04 Gbp (Jakob *et al*., 2004; Doležel *et al*., 2018).

**Fig. 2:**
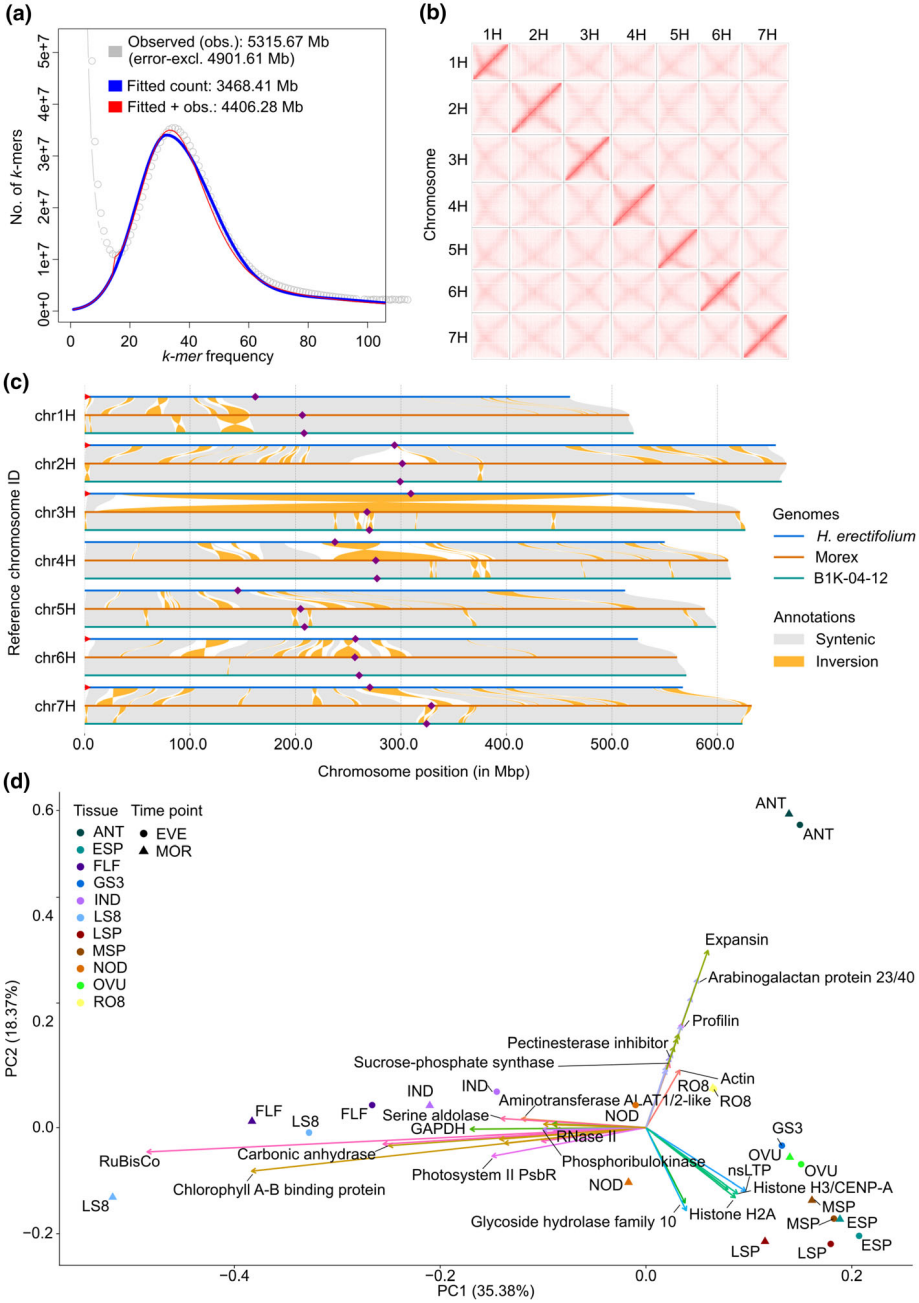
Genome characteristics, interchromosomal contact matrix, chromosomal synteny between genomes, and principal component analysis (PCA) of tissue‐specific expression profiles. (a) *k*‐mer spectra of 21‐mers, calculated from short‐read data, the red line indicates fitted and observed 21‐mers for genome size calculation. (b) Interchomosomal contact matrix of the seven assembled chromosomes. Pixel intensity represents Hi‐C link counts normalized over a 1 Mb window on a logarithmic scale. (c) Whole chromosomal synteny between each respective chromosome in *Hordeum erectifolium*, Morex and B1K‐04‐12. Inversions smaller than 1 Mb were excluded for better visualization. Purple diamonds indicate putative centromere location, and red triangles indicate identified telomeric sequences. (d) PCA clustering of transcript abundance in 22 tissue‐time specific samples and the trajectory of the top 20 highest loading genes for PC1 and PC2. Eleven individual tissues, of which 10 were sampled both in the morning (MOR, ZT 1–3) and evening (EVE, ZT 13–15). Opposite trajectories of vegetative vs reproductive tissues along PC1 and a third trajectory on PC2 contributed by anthers (ANT) and, to a lesser extent, roots (RO8). Flag leaf (FLF), 3‐d‐old germinating seeds (GS3), third internode (IND), fourth node (NOD), whole shoots (LS8, 8 d post germination), whole roots (RO8, 8 d post germination), ovules (OVU), anthers (ANT), caryopses (CAR), developing shoot apical meristems: ESP (W3.0–4.5), MSP (W5.0–6.5), LSP (W7.0–8.0) (Waddington *et al*., 1983).

Using short‐read sequencing, 10× Genomics Linked‐Reads, we characterized the haploid genome of *H. erectifolium* via *k*‐mer analysis, estimating a genome size of 4.4 Gbp, a repeat content of 75%, and no detectable heterozygosity (Fig. 2a). For a high‐quality chromosome‐scale genome assembly, we used a combination of ONT long (321 Gb, 72× coverage, read N50 of 42 kb), and Illumina short (244.6 Gb, 55.6× coverage) read data, Bionano optical genome mapping (546 optical genome maps, map length of 3.8 Gbp, N50 of 20.1 Mbp) for hybrid scaffolding and Hi‐C chromosomal conformation capture sequencing for pseudomolecule construction (72.2 Gb, 16.4× coverage) (Tables S1–S4). The final assembly size was 3.94 Gbp, with 3.85 Gbp (97.7%) being anchored in seven pseudomolecules and 89 Mbp of unassigned contigs. (Fig. 2b,c; Table S5). The genome had a *k*‐mer completeness score of 94% and consensus quality (QV) estimate score of QV 34.6, and a BUSCO estimate of gene space completeness of 98.2% (Single: 93.4%, Duplicate: 4.8%, Poales database, *n* = 4896) (Table S5) (Rhie *et al*., 2020; Manni *et al*., 2021).

Taken together, we assembled a high‐quality chromosomal‐scale reference genome of *H. erectifolium* using ONT long reads and polished with Illumina short reads, attaining a consensus score of QV 34.6. The final genome consisted of 3.85 Gbp assigned to 7 pseudomolecules.

### Tissue‐time‐specific long‐read transcriptome sequencing and gene annotation

Transcriptome profiling of 22 samples from 12 different tissues harvested at two diurnal time points, morning (MOR) and evening (EVE) yielded 3 645 621 full‐length and nonchimeric reads used for annotation and analyses (Tables S6, S7). PCA grouped the MOR and EVE tissue pairs closely together, vegetative and reproductive samples differentiated along PC1 (35%) with roots (RO8) and nodes (NOD) at the center. High expression of PROLIFIN and EXPANSIN genes separated ANT from other reproductive samples (Fig. 2d). When the CAR was included, it accounted for 49% of the PC1 variance, primarily due to high expression of multiple *GLIADIN* genes, which encode the major storage proteins in cereal grains (Fig. S2) (Anderson *et al*., 2012). The trajectories of developing shoot apical meristem (ESP, MSP, LSP) and ovules (OVU) were most influenced by histones (*H2A* and *H3/CENP‐A*) and a nonspecific lipid‐transfer protein (*nsLTP*). Photosynthesis‐related genes, particularly Chl*a*‐*b* binding protein and Ribulose‐1,5‐bisphosphate carboxylase/oxygenase, were the main drivers distinguishing aboveground vegetative samples, including whole shoots (LS8) and flag leaves (FLF; Figs 2d, S2). We combined two structural gene annotation methods, in which IsoSeq yielded 29 099 protein‐coding genes (including 5′ and 3′ untranslated regions (UTR)) and 8993 putative lncRNAs, Helixer predicted 55 287 protein‐coding genes, of which 20 875 fully and 1029 partially overlapped with IsoSeq. We annotated a total of 71 475 genes, of which 8993 are lncRNAs. The use of IsoSeq sequencing provided additional information on 79 149 unique protein‐coding isoforms, with 3.4 isoforms per gene, 5.9 exons per mRNA and 4.5 exons per coding sequence (CDS), respectively. The total gene and CDS mean lengths were 3292 and 987 bp, respectively, whereas high confidence (HC) genes, 40 035, had respective gene and CDS mean lengths of 3894 and 1257 bp. The lncRNA genes were considerably shorter with a mean length of 1857 bp, and only 1.8 isoforms and two exons per gene (Table S8).

In summary, using IsoSeq mRNA sequencing of 22 tissue samples, we generated high‐accuracy gene predictions with UTRs and accurate isoform information, as well as lncRNA predictions. Complemented with *ab initio* gene prediction, we identified and annotated a final set of 71 475 genes (thereof 8993 lncRNAs) and 157 682 isoforms. Furthermore, we provided a comprehensive tissue‐ and time‐of‐day‐specific gene expression atlas in *H. erectifolium*.

### Comparative genome analysis between *H. erectifolium* and *H. vulgare*

We further characterized the genome of *H. erectifolium* in relation to the reference genomes of the spring barley cultivar Morex and the wild barley accession B1K‐04‐12 (Jayakodi *et al*., 2020; Mascher *et al*., 2021). We compared the chromosome organization and structure, repeat content and identified telomeric sequences, centromere locations and TE composition.

The assembly of telomeres and centromeres is one of the remaining challenges in gapless genome assemblies, due to the presence of long stretches of satellite repeats (Navrátilová *et al*., 2022). Here, we report that the telomeric ends were only found on the short arms of five of seven chromosomes (missing on 4H and 5H) in *H. erectifolium* (Fig. 2c). No telomeric ends were found on long and short arms in either of the two barley genomes. We found *CRM* (homolog of *Cereba* in barley) enrichments in the pericentromeric regions spanning on average *c*. 37 Mbp per chromosome in *H. erectifolium*, and *c*. 100 intact *CRMs* per chromosome, but only *c*. 18 intact in barley (Figs 2c, S3) (Presting *et al*., 1998; Hudakova *et al*., 2001; Neumann *et al*., 2011). The *CRM* enrichment midpoints also colocalized with the regions identified in the Hi‐C matrices (Fig. 2b) (Navrátilová *et al*., 2022).

Large‐scale chromosomal synteny among *H. erectifolium*, Morex, and B1K‐04‐12 was overall conserved, although numerous small‐ to medium‐scale structural rearrangements were detected. However, two notable large pericentric inversions were found, a near full chromosomal inversion on 3H that spanned 471 Mbp (81.4%) in *H. erectifolium* and 570 Mbp (91.7%) in Morex, and on 4H a pericentric inversion of 56 Mbp (10.2%) in *H. erectifolium* and 109 Mbp (17.9%) in Morex (Fig. 2c). Neither inversion had previously been reported in barley (Jayakodi *et al*., 2024; Mascher *et al*., 2024). Between *H. erectifolium* and Morex, we found 59 inversions > 1 Mbp, and 17 inversions > 10 Mbp and thus more than between Morex and B1K‐04‐12, with 32 inversions > 1 Mbp and one inversion > 10 Mbp.

**Fig. 3:**
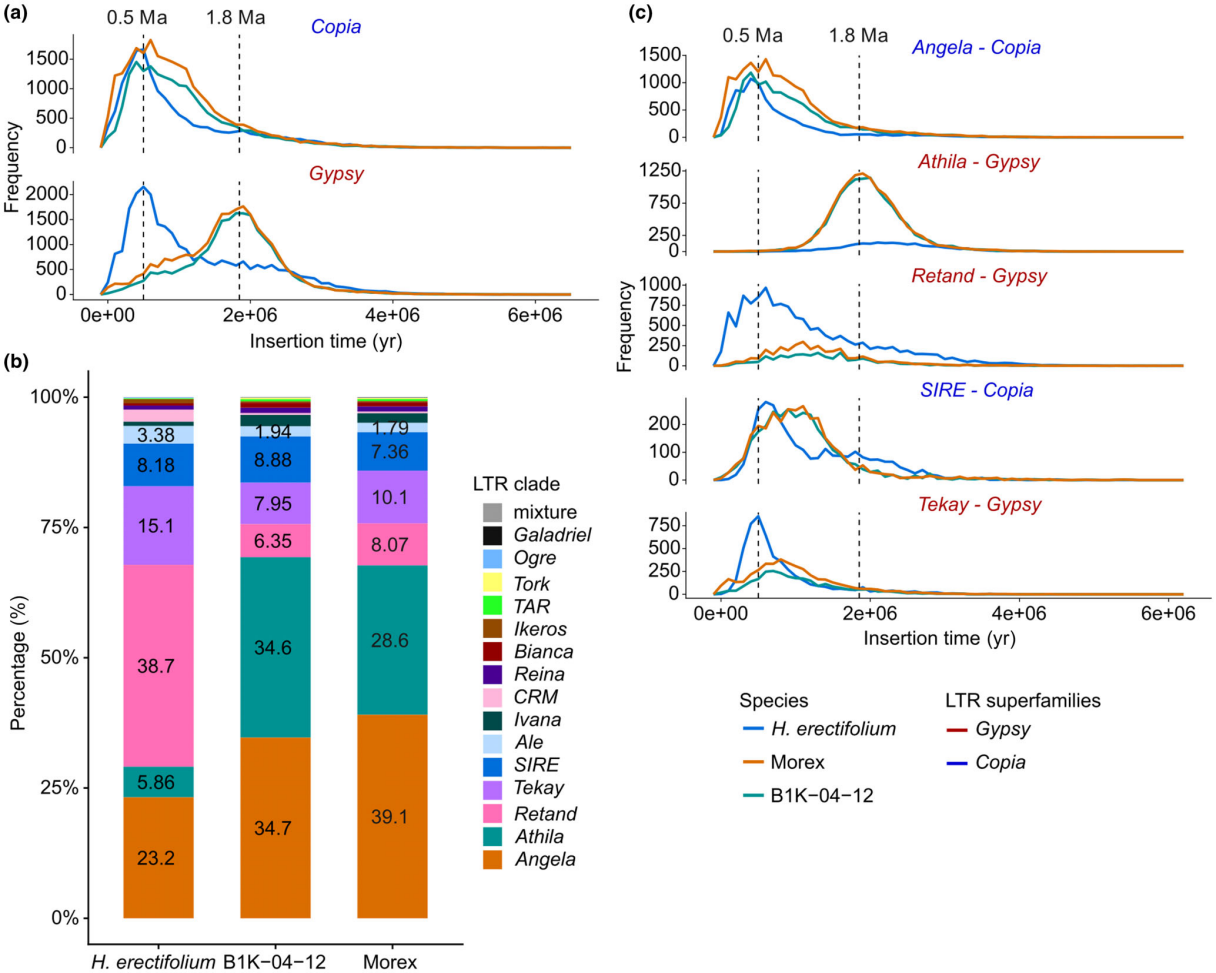
Transposable elements composition, long terminal repeat (LTR) retrotransposon profiles and frequency of predicted insertion times. (a) Frequency plot of estimated LTR retrotransposon superfamily insertion times, *Gypsy* (red) and *Copia* (blue). Insertion times were calculated based on the mutation rate in rice (1.3 × 10^−8^) of intact LTR retrotransposons in *Hordeum erectifolium*, Morex, and B1K‐04‐12. Dotted lines mark LTR retrotransposon insertion burst peaks at 0.5 million years ago (Ma) and *c.* 1.8 Ma. (b) Composition landscape of intact LTR retrotransposons in *H. erectifolium*, Morex and B1K‐04‐12. The percentage of LTR retrotransposons was normalized to the total of intact LTR retrotransposons within a genome. (c) Intact LTR retrotransposon insertion frequency over time by clade for the five most abundant clades; *Copia*: *Angela* and *SIRE* (blue), *Gypsy*: *Athila*, *Retand*, and *Tekay* (red). The black dotted lines mark 0.5 and 1.8 Ma, respectively.

We performed *de novo* annotation of TEs in the *H. erectifolium* genome and re‐annotated TEs in the barley genomes Morex and B1K‐04‐12. TE content was relatively consistent across genomes, covering 85.7% in *H. erectifolium*, 87.5% in Morex and 86.9% in B1K‐04‐12. In *H. erectifolium*, the majority of repeats were LTR retrotransposons (68.2%), while non‐LTR elements included terminal inverted repeats (11.3%) and Helitrons (5.3%) (Table S9). Among LTRs, *Gypsy* elements were most abundant (20.1%), followed by *Copia* (11.5%), with 37.6% classified as mixed or unclassified LTRs. Notably, CACTA transposons were enriched in *H. erectifolium* (7.2%) but were less prevalent in Morex (1.3%) and B1K‐04‐12 (2.2%) (Table S9). Although overall LTR content was comparable across genomes, the number of intact LTRs varied by clade (Fig. 3b). In *H. erectifolium*, *Retand* (39%), *Angela* (23%), *Tekay* (15%) and *SIRE* (8%) dominated, accounting for 85% of all intact LTRs. By contrast, Morex and B1K‐04‐12 were primarily composed of *Angela* (39%, 35%), *Athila* (29%, 35%), *Tekay* (10%, 8%) and *SIRE* (7%, 9%), contributing 85% and 87% of their LTR content, respectively.

Insertion times of intact LTRs were estimated using a nucleotide substitution rate of 1.3 × 10^−8^ to infer their activation periods (Ma & Bennetzen, 2004; Ou & Jiang, 2018). We observed two distinct *Gypsy* insertion peaks: one at *c*. 0.5 Ma in *H. erectifolium*, and another at *c*. 1.8 Ma in Morex and B1K‐04‐12. *Copia* showed a concurrent insertion burst *c*. 0.5 Ma in all three genomes during the Chibanian, coinciding with intensified and asynchronous glacial cycles (Fig. 3a) (Sun *et al*., 2021). At this time, *Copia* insertions were predominantly driven by the *Angela* clade across all genomes, while *H. erectifolium* also showed lineage‐specific bursts of the *Gypsy* clades *Tekay* and *Retand* (Fig. 3c). The older burst at *c*. 1.8 Ma, unique to Morex and B1K‐04‐12, involved *Athila* insertions, corresponding with the Gelasian–Calabrian transition and early Northern Hemisphere glaciation (Gibbard *et al*., 2010; Cita *et al*., 2012). Recent *Copia* insertions were more frequent near chromosomal ends in all genomes, although they were also dispersed across chromosomes. Additionally, in *H. erectifolium*, *Gypsy* insertions exhibited a similar distribution pattern, recent (< 0.6 Ma) and older (> 0.6 Ma) (Fig. S4).

In summary, we identified numerous small‐ to medium‐scale structural rearrangements between *H. erectifolium* and barley, along with two large‐scale pericentric inversions on chromosomes 3H and 4H. Despite similar TE amounts, each species displayed distinct LTR retrotransposon profiles, indicating independent TE‐driven genome evolution. Additionally, we detected two major LTR insertion bursts across the *Hordeum* genus, occurring *c*. 0.5 and 1.8 Ma, coinciding with significant geological and climatic events.

### Genetic signatures of adaptive abiotic stress‐related gene family expansions in *H. erectifolium*

We established HOGs across the Poaceae family: *Sorghum bicolor Zea mays*, *Oryza sativa*, *Brachypodium distachyon*, *Triticum aestivum*, *Thinopyrum intermedium* and *H. vulgare*, along with *Arabidopsis thaliana* as an outgroup, to explore unique or shared gene families, expansions, or contractions. *Th. intermedium* was added as another perennial stress‐tolerant species.

**Fig. 4:**
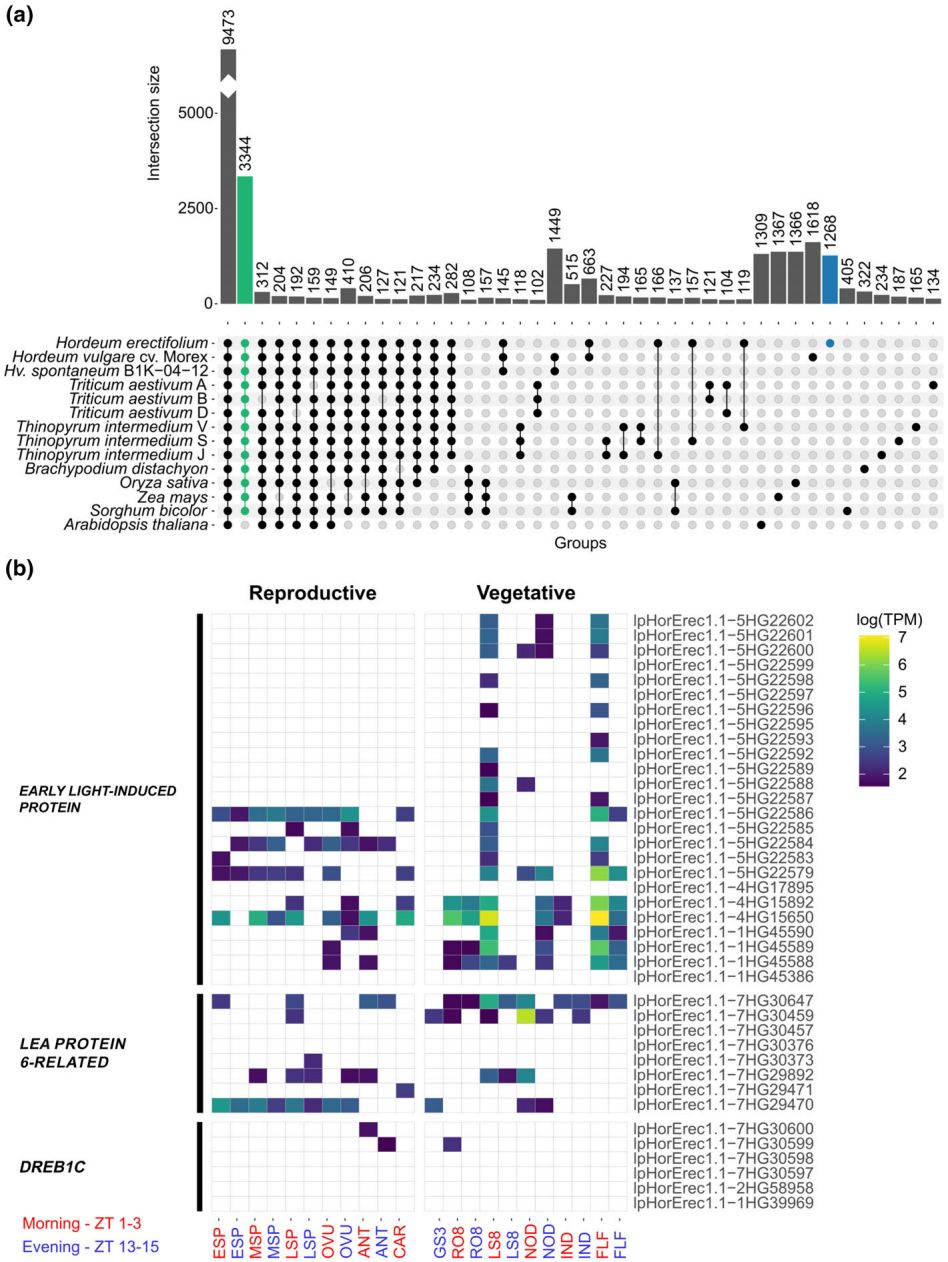
Shared and unique orthologs of *Hordeum erectifolium* with Poaceae and *Arabidopsis thaliana*. Tissue and time‐specific expression of expanded gene families. (a) Shared and unique hierarchical phylogenetic orthologs (HOGs) between nine genomes of eight species; eight Poaceae species plus *A. thaliana*, the wheat (*Triticum aestivum*) (A, B and D) and *Thinopyrum intermedium* (J, S and V) subgenomes were separated. Shared HOGs in Poaceae (green) and unique to *H. erectifolium* (blue) (b) Heatmap with expressed genes of expanded gene families found in the 22 tissue‐and‐time specific samples; *EARLY LIGHT‐INDUCED PROTEINS* (*ELIP*) (N0.HOG0000166), *LATE EMBRYOGENESIS ABUNDANT PROTEIN 6‐RELATED* (*LEA PROTEIN 6‐RELATED*) (N0.HOG0000799), and *DEHYDRATION‐RESPONSIVE ELEMENT‐BINDING PROTEIN 1C* (*DREB1C*) (N0.HOG0001187). Transcript abundance indicated as log(TPM), and timepoints: morning (red, MOR, ZT 1–3) and evening (blue, EVE, ZT 13–15). Tissues are divided into reproductive: developing shoot apical meristems: ESP (W3.0–4.5), MSP (W5.0–6.5), LSP (W7.0–8.0), ovules (OVU), anthers (ANT), caryopses (CAR), and vegetative: 3‐d‐old germinating seeds (GS3), whole roots (RO8, 8 d post germination), whole shoots (LS8, 8 d post germination), fourth node (NOD), third internode (IND), and flag leaf (FLF) (Waddington *et al*., 1983).

We identified 38 190 HOGs, 9473 of which were shared across the Poaceae and Arabidopsis and 3344 exclusive to Poaceae, while 1268 HOGs were unique to *H. erectifolium* (Fig. 4a). There were 6110 genes in the 1268 HOGs unique to *H. erectifolium* with GO enrichments related to biological functions such as methylation, ribosome biogenesis, cell redox homeostasis and signal peptide processing (Fig. S5a).

We postulated that *H. erectifolium* and *Th. intermedium* might be characterized by similar adaptive copy number variation (CNV) and therefore tested HOG expansion and contraction in *H. erectifolium* and *Th. intermedium* separately. The number of expanded and contracted HOGs evaluated for significance did not greatly vary, 19 110 with *H. erectifolium* and 19 182 with *Th. intermedium* (Fig. S6; Table S10).

In *H. erectifolium*, we identified 902 HOGs significantly expanded or contracted, 808 expansions and 94 contractions (Table S11). The 5941 genes from *H. erectifolium* belonging to the 902 HOGs were enriched for terms related to anatomical structure development, cell surface receptor signaling and root development (Fig. S5b). Of those, we selected three expanded HOGs, with putative functions in desiccation tolerance that had undergone expansions in *H. erectifolium* (Fig. S7; Table S12).

The *EARLY LIGHT‐INDUCIBLE PROTEIN* (*ELIP*, N0.HOG0000166) gene family, which enhances desiccation tolerance by mitigating photooxidative damage (VanBuren *et al*., 2019), is expanded in *H. erectifolium* with 25 paralogs, compared to 20 in barley (Fig. S7a). The primary expansion occurred through tandem duplication on chromosome 5H, with 18 copies in *H. erectifolium* and 12 in barley. Of the 25 *ELIP* genes, 20 showed tissue‐ and time‐specific expression: 11 were exclusively expressed in vegetative tissue, predominantly in leaves in the morning (LS8, FLF), whereas nine were expressed both in vegetative and reproductive tissues (Fig. 4b; Table S13). We also observed an expansion of *LATE EMBRYOGENESIS ABUNDANT* (*LEA*, N0.HOG0000799) *PROTEIN 6‐RELATED* genes (group 4 LEAs), which protect proteins and membranes during desiccation (Candat *et al*., 2014). *H. erectifolium* carried eight paralogs vs four in barley, with six showing expression in at least one tissue or time point (Figs 4b, S7b; Table S13). Similarly, the *DEHYDRATION‐RESPONSIVE ELEMENT‐BINDING PROTEIN 1C* (*DREB1C*, N0.HOG0001187) family underwent tandem duplication on chromosome 7H in *H. erectifolium*, resulting in four paralogs compared to two in barley; however, only two were expressed in the IsoSeq data, each in a single tissue (Figs 4b, S4c; Table S13).

In *Th. intermedium*, we identified in the subgenomes; 1326 J, 691 S, 582 V, HOGs that were significantly expanded or contracted, 891 J, 578 S, 452 V, expansions, and 435 J, 113 S, 130 V, contractions (Tables S10, S11). *Th. intermedium* also showed significant expansion of LEA, ELIP and DREB1C HOGs (Fig. S7d–f). Interestingly, these copy number expansions originated from different chromosomes in *H. erectifolium* and *Th. intermedium* and differed between homologous chromosomes within the hexaploid *Th*. *intermedium*. For example, in *H. erectifolium*, tandem duplication expansions of DREB1C occurred on chromosome 7H, but on chromosome 2 in *Th. intermedium*, with four copies on 2S and 2J and seven copies on 2V.

In summary, gene sets unique to *H. erectifolium* were enriched for functions including methylation, ribosome biogenesis, cell redox homeostasis and signal peptide processing. HOGs expanded in *H. erectifolium* were enriched for terms related to anatomical structure development, cell surface receptor signaling and root development. The *H. erectifolium* genome was characterized by significant gene copy expansions, that is for desiccation tolerance genes, a pattern also observed in the stress‐tolerant perennial *Th. intermedium*.

### Cross‐species transcriptional drought response and recovery

To explore differences and similarities in the transcriptional responses to drought between *H. erectifolium* and barley cv Morex, we set up a drydown and recovery experiment at the vegetative stage. Using a controlled drydown assay, we ensured an equal reduction in the soil's FC, thereby achieving a comparable reduction in the leaf RWC between plants of the two species.

During drydown, both leaf RWC and soil FC decreased at the same rate in both species, with no significant differences between either species at each respective DOT time point (Figs S8, S9). Control samples were kept at 50% soil FC while water was withheld for 6 d before rewatering, and samples were collected on Days 2, 5, 6 and 7 of treatment. On the last day of drydown, DOT6, the average leaf RWC and soil FC for *H. erectifolium* were at 42% ± 9% and 11% ± 2%, and for Morex at 51% ± 9% and 13% ± 0.6%, respectively. Strong phenotypic differences at DOT5 and DOT6 were observed; leaves of *H. erectifolium* had rolled inward and remained erect, whereas Morex leaves wilted and required structural support (Fig. S8b,c). Both species recovered leaf RWC to control levels and regained leaf structure on DOT7, 24 h after rewatering. No visible symptoms of senescence, that is, no yellowing of leaves, were observed at DOT7 nor 7 d after rewatering, DOT13.

In total, 64 leaf samples from the two species, four time points and two treatments with four biological replicates per sampling point were RNA‐sequenced with an average of 21 million reads per sample (Table S14). The RNAseq data were mapped to their respective genomes with STAR and quantified with featureCounts (Table S15) (Dobin *et al*., 2013; Liao *et al*., 2014). We employed two methods for transcriptomic analysis: maSigPro for time‐course analysis, taking into account day of sampling and treatment variables (Nueda *et al*., 2014), and edgeR for pairwise expression comparison between control and drydown for each time point separately to also identify transiently expressed genes (Chen *et al*., 2025).

With both methods, we identified 15 036 and 13 119 DEGs in *H. erectifolium* and Morex, respectively (Tables S16–S18). The time‐course analysis identified 7941 DEGs in *H. erectifolium* and 7382 DEGs in Morex (Fig. S10). In addition, we detected 7095 DEGs in *H. erectifolium* and 5737 DEGs in Morex, which were expressed only at specific DOTs and not identified in the time‐course analysis.

**Fig. 5:**
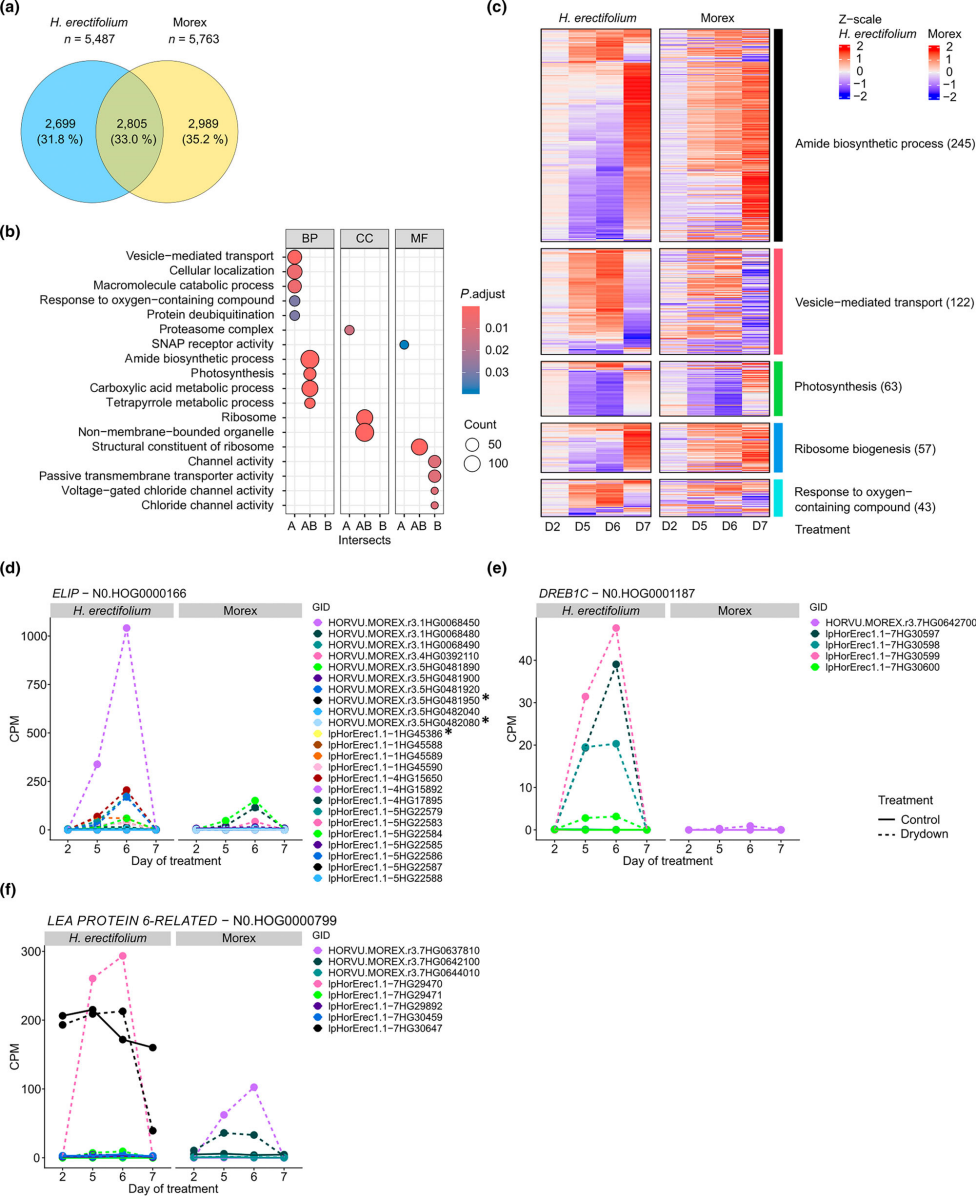
Differentially expressed genes (DEG) during drydown and recovery in *Hordeum erectifolium* and Morex. (a) Total number of shared and exclusive single‐copy ortholog (SCO) genes differentially expressed between *H. erectifolium* and Morex. (b) Gene Ontology (GO) enrichment of shared and exclusive SCO DEGs: exclusive to *H. erectifolium* (A), exclusive to Morex (B), and interspecies overlap (AB). GO categories: biological processes (BP), cellular compartments (CC), and molecular functions (MF). (c) Five groups of the enriched biological processes terms in SCO: amide biosynthetic process, vesicle‐mediated transport, photosynthesis, ribosome biogenesis, and response to oxygen – containing compounds, total number of genes shown in brackets. The heatmap shows scaled log(fold‐change), and gene clustering was based on *H. erectifolium* transcripts with the corresponding SCO in Morex fixed to the same location in the heatmap. Second day‐of‐treatment (DOT) (D2); fifth DOT (D5); D6, sixth DOT (D6); seventh DOT (D7, recovery). Gene expression of expanded hierarchical phylogenetic orthologs (HOG) gene families in *H. erectifolium* and Morex genes found in the same HOG in response to drydown and recovery: (d) *EARLY LIGHT‐INDUCED PROTEINS* (*ELIP*) (N0.HOG0000166), (e) *DEHYDRATION‐RESPONSIVE ELEMENT‐BINDING PROTEIN 1C* (*DREB1C*) (N0.HOG0001187), (f) *LATE EMBRYOGENESIS ABUNDANT PROTEIN 6‐RELATED* (*LEA PROTEIN 6‐RELATED*) (N0.HOG0000799). Graphs show normalized counts per million values for control and drydown. All genes, except those marked with an asterisk, were differentially regulated in response to drydown or recovery.

To compare DEGs between the two species, we focused on their shared SCO. Since transient DEGs showed a low overlap between species, we only included DEGs identified by the time‐course analysis. Between *H. erectifolium* and Morex, we identified 19 204 SCO, of which 8451 were differentially expressed in at least one species, of them 5487 in *H. erectifolium* and 5743 in Morex, with an overlap of 2799 (33%) DEGs detected in both *H. erectifolium* and Morex (Fig. 5a; Table S19).

DEGs shared by both species were enriched for photosynthesis, translation (ribosome), amide biosynthesis, carboxylic acid and tetrapyrrole metabolism, suggesting that both species adapt metabolically and coordinate energy production, carbon and nitrogen assimilation and oxidative stress management in response to water limitations (Fig. 5b). However, while both species downregulated genes with functions in photosynthesis during water limitation, they exhibited markedly different responses in genes related to biosynthesis and translation. Notably, DEGs associated with biosynthesis (amide biosynthetic processes) and translation (ribosome‐related processes) were strongly downregulated in *H. erectifolium*, whereas they were upregulated in Morex at DOT5 and DOT6 (Fig. 5c). In *H. erectifolium*, the downregulation of these DEGs indicated a repression of biosynthesis and translational activity in response to drought. The downregulation of amide biosynthesis, that is decreased transcript levels of glutamine and asparagine synthase genes (Tables S17, S18), likely reflected a metabolic shift toward energy conservation to prioritize survival over growth. The concurrent upregulation of ubiquitination, macromolecule catabolism and vesicle‐mediated transport specifically in *H. erectifolium* indicated mobilization of proteins for recycling or stress adaptation and active degradation of damaged proteins and cellular components. By contrast, Morex upregulated genes involved in amide biosynthesis and translation, potentially as part of a protective response to counteract reactive oxygen species (ROS)‐induced protein denaturation and to replace damaged proteins to maintain cell functions. The transcriptional responses in Morex were thus reminiscent of proteostasis responses, which commonly lead to massive transcriptional and translational upregulation of protective proteins (Mittler *et al*., 2012). The Morex‐specific enrichment of DEGs with functions in transmembrane transport and chloride channel activities suggested that Morex maintained ion and osmotic balance to support active metabolism under water limitations. Morex thus invested in maintaining cellular functions and supporting future recovery rather than shutting down. In line with this, at recovery (DOT7), transcripts related to biosynthesis and translation were strongly induced in *H. erectifolium* but not strongly altered compared to DOT5 and 6 in Morex (Fig. 5c).

Given the different transcriptional responses to water limitation between both genotypes, we further explored the expression of the three expanded desiccation tolerance gene families, encoding *DREB*, *ELIP* and *LEA* proteins, which function in cellular protection and stabilization, particularly during dehydration or oxidative stress. Among the *ELIP* (N0.HOG0000166) genes, 13 of 25 and eight of 20 were differentially expressed during desiccation in *H. erectifolium* and Morex, respectively (Figs 5d, S11a). We observed a strong upregulation of the four *DREB1C* genes (N0.HOG0001187) in *H. erectifolium* compared to only a mild upregulation of one *DREB1C* copy in Morex (Figs 5f, S11b). *DREB* transcription factors act as upstream regulators that activate *LEA* genes, which were also more strongly expressed in *H. erectifolium* than in Morex (Sakuma *et al*., 2006). Of the *LEA* (N0.HOG0000799) genes, five of the eight copies were upregulated in *H. erectifolium*, whereas only three of four copies were upregulated in Morex in response to drought (Figs 5e, S11c). One *LEA* (lpHorErec1.1‐7HG30647) had a high constitutive expression under control and drought in *H. erectifolium* and was also expressed in nearly all vegetative tissues (Fig. 4b).

Taken together, the differential regulation of amide biosynthesis and translation in *H. erectifolium* and Morex suggested contrasting metabolic responses to water limitations. Morex upregulated both pathways, thereby maintaining active nitrogen metabolism, detoxification and proteostasis. By contrast, *H. erectifolium* downregulated both pathways, indicative of a conservative, survival‐focused strategy, minimizing energy expenditure and growth processes to preserve cellular integrity during drought. The expanded and highly inducible *DREB*, *LEA* and *ELIP* families suggested that *H. erectifolium* is genetically primed for drought, enabling strong protective responses with low metabolic cost.

## Discussion

### The unique leaf morphology of *H. erectifolium*

*Hordeum erectifolium* displayed distinct leaf morphological traits indicative of drought adaptation, including dense trichome coverage, high vein density with a near‐equal major‐to‐minor vein ratio and a thick cuticular wax layer, all features of xeromorphic leaf traits (Shields, 1950). The prominently ribbed adaxial surface bore dense short trichomes and longer trichomes along major veins, features involved in microclimate stabilization, dew capture and enhanced structural rigidity (Liakoura *et al*., 1997; Galdon‐Armero *et al*., 2018). A compact, uniform epidermal cell layer and a substantial wax coating likely strengthened the transpiration barrier, UV protection and prevented biotic intrusion (Kasapligil, 1961; Xue *et al*., 2017). Major veins were reinforced with extensive BSE, sclerenchymatous tissue causing increased mesophyll compartmentalization, which has been associated with enhanced light penetration, photosynthetic efficiency and water‐use optimization through more responsive BSE‐connected stomatal regulation (Barbosa *et al*., 2019). *H. erectifolium* and Morex both maintained comparable leaf RWC levels as soil moisture declined during drydown. However, while Morex leaves wilted and required external support, *H. erectifolium* leaves rolled tightly and remained erect (Figs 1, S8). Leaf rolling under water deficit reduces the exposed surface area, thereby minimizing transpiration, photodamage and heat stress (Kadioglu *et al*., 2012). Although bulliform cells are typically associated with this response, they were largely absent in *H. erectifolium* (Cal *et al*., 2019; Zhu *et al*., 2022). We propose that leaf rolling in this species is facilitated by the ribbed structure, numerous major veins with extensive BSE and sclerenchyma, and a compact epidermal cell architecture (Guo *et al*., 2024). The distinctive morphology of *H. erectifolium* was reflected at the genomic level by an enrichment of expanded gene families involved in the development of anatomical structures.

### Clade‐specific LTR retrotransposon insertions in *H. erectifolium* and barley

Triticeae genomes are typically large (*c*. 5 Gbp per genome or subgenome) and consist of up to 90% repetitive elements, primarily TEs (Middleton *et al*., 2013; Winterfeld *et al*., 2025). While the total proportion of repetitive content was relatively stable across species (85.7%–87.5%), we observed notable differences in LTR clade composition and insertion histories. *H. erectifolium* showed a fivefold enrichment of CACTA transposons compared to barley, elements that influence genome restructuring and gene evolution (Lisch, 2013; Catoni *et al*., 2019; Liu *et al*., 2020). We discovered two LTR retrotransposon insertion bursts at *c*. 0.5 Ma and *c*. 1.8 Ma, which coincide with known geological events. The Chibanian stage (*c*. 0.5 Ma), characterized by intense, globally asynchronous glacial cycles, and the Calabrian stage (Early Pleistocene, *c*. 1.8 Ma), marked by the onset of Northern Hemisphere glaciation affecting Eurasian species (Cita *et al*., 2012; Sun *et al*., 2021). There was a synchronous peak at *c*. 0.5 Ma, where *Angela* (*Copia*) was active in both species, while the *Gypsy* families *Retand* and *Tekay* were specific to *H. erectifolium*. The second unilateral peak at *c*. 1.8 Ma, which was observed only in barley, was exclusively associated with *Athila* (*Gypsy*) (Fig. 4). LTR insertion bursts 1.8 Ma were also detected in two other Mediterranean species, wheat and *Brachypodium distachyon* (Wicker *et al*., 2018; Stritt *et al*., 2020), suggesting that Eurasian species experienced more severe glaciation and biome turnover than South American species (Vuilleumier, 1971; Connor, 2009; Gibbard *et al*., 2010). Accordingly, phylogenetic and biogeographic studies suggested high extinction rates for the Eurasian *Hordeum* species during the Early Pleistocene, while high speciation rates were maintained in the South American clade (Jakob & Blattner, 2006).

### Tandem duplication of desiccation‐responsive genes and activation during drydown

The higher copy number of desiccation‐related genes and the distinct transcriptome response to water limitation indicated that *H. erectifolium* and Morex diverge not only in their morphological but also in their molecular responses to stress. Gene family expansion through tandem duplication can enable faster transcriptional activation and greater dosage effects, providing an adaptive advantage during rapid declines in RWC (Dassanayake *et al*., 2011; Wu *et al*., 2012). Large tandem arrays of *ELIP*s, which stabilize and protect photosynthetic components during dehydration and enhance thermal energy dissipation, have been repeatedly associated with independent origins of desiccation tolerance across diverse plant lineages (VanBuren *et al*., 2019; Pardo *et al*., 2020; Marks *et al*., 2024). Similarly, the observed expansion of *DREB1C* and *LEA* gene families in *H. erectifolium* is consistent with a genetic architecture that enhances resilience across multiple stresses, including drought, salinity and cold (Hernández‐Sánchez *et al*., 2022; Wang *et al*., 2022). Such expansions likely genetically primed *H. erectifolium* for rapid adaptation to fluctuating water availability, offering a selective advantage in environments where desiccation events are frequent and unpredictable. This genetic priming for rapid stress response is reminiscent of mechanisms seen in resurrection plants, where tandem gene duplications of protective proteins underpin efficient tolerance with minimal metabolic burden (VanBuren *et al*., 2019; Pardo *et al*., 2020). The drydown experiment corroborates this hypothesis, showing fast and strong activation of all three gene families followed by rapid deactivation upon rehydration, a pattern indicative of efficient regulatory control. Furthermore, the constitutive high expression of one *LEA* copy may have conferred an immediate protective buffer at the onset of dehydration stress, minimizing cellular damage during early water loss (Cheng *et al*., 2002; Liu *et al*., 2009).

### Transcriptional responses to water limitation suggest different drought adaptation strategies in *H. erectifolium* and barley

The transcriptional responses of *H. erectifolium* and Morex to water limitation suggested contrasting drought‐adaptation strategies: metabolic downregulation and survival prioritization in *H. erectifolium* vs maintenance of metabolic activity and competitiveness in Morex (Skirycz *et al*., 2011; Claeys & Inzé, 2013). Both genotypes showed enrichment of DEGs related to photosynthesis, translation and nitrogen metabolism, but their regulation differed markedly. In *H. erectifolium*, strong repression of amide biosynthesis and ribosome‐related processes, including reduced expression of glutamine and asparagine synthetases, indicates energy conservation through suppression of growth and nitrogen assimilation (Díaz *et al*., 2010; Nagy *et al*., 2013). The concurrent induction of ubiquitination and macromolecule catabolism suggests active recycling of proteins and degradation of damaged components to protect cellular integrity (Skirycz & Inzé, 2010; Eckardt *et al*., 2024). Furthermore, *H. erectifolium* exhibited unique activation of pathways linked to cellular localization, vesicle‐mediated transport and oxidative stress mitigation, including ROS metabolism, which together indicate pre‐emptive preparation for drought (Mazel *et al*., 2004; Jarzyniak & Jasiński, 2014; Noctor *et al*., 2014). By contrast, Morex upregulated biosynthesis and translation, consistent with a strategy of sustaining metabolic activity and proteostasis to replace damaged proteins and maintain cellular function (Mittler *et al*., 2012). The enrichment of transmembrane transport and chloride channel activity indicates active osmotic regulation, supporting growth and recovery under moderate stress (Nieves‐Cordones *et al*., 2019; Franco‐Navarro *et al*., 2021). The absence of strong postdrought induction of biosynthetic and translational processes in Morex at recovery, compared to the rapid reactivation seen in *H. erectifolium*, further highlights differences in resource allocation and stress recovery dynamics.

Overall, *H. erectifolium* appears genetically primed for survival under severe drought through gene family expansion and the rapid induction of protective responses, efficient shutdown of growth‐related metabolism and rapid recovery. Such a strategy is likely advantageous in habitats with frequent and severe desiccation events, favoring survival over productivity. Morex, by contrast, invests in maintaining metabolic activity during drought, which may favor productivity and recovery potential under mild stress but is energetically costly under prolonged or severe water deficits (Skirycz *et al*., 2011).

Combining both strategies, stress avoidance through active metabolism with drought tolerance via growth suppression, is a central challenge for breeding crops that can both survive severe drought and maintain yield under moderate stress. The morphological and molecular adaptations of *H. erectifoliu*m could be leveraged to enhance drought tolerance in barley. Specifically, *H. erectifolium* provides leads for modifying leaf characters, such as vein density and the production of cuticular waxes, which influence water transport and diffusion from the leaf (Tabassum *et al*., 2016; Hasanuzzaman *et al*., 2017). Candidate genes for these processes identified in rice and barley will support the identification of genetic variants, regulatory and coding, underlying these traits in *H. erectifolium* (Liu *et al*., 2023; Campoli *et al*., 2024). Furthermore, copy number variation and the tissue‐specific expression of different LEA, DREB1C and ELIP copies could be exploited to fine‐tune tissue‐specific desiccation responses. Engineering tissue and stage‐specific expression of stress responses could be an approach to improve tolerance while maintaining growth. Taken together, information from *H. erectifolium*, but also *Th. intermedium* can be exploited to engineer copy number variation and stress‐responsive promoter elements to activate key protective genes while minimizing growth penalties under transient or moderate stress in barley and wheat (Nelson *et al*., 2007; Peleg *et al*., 2011).

## Competing interests

None declared.

## Author contributions

EBH and MvK conceived and designed the project and experiments. EBH performed all plant growth, DNA extractions, data processing, genome assembly, experiments and analysis, with the following exceptions: HŠ, HT and ZT made the optical genome map and did the hybrid scaffolding. MA provided the carbon‐nitrogen elemental and Specific Leaf‐Area measurements. TR performed RNA extractions for sequencing. MM and J‐WF helped with the chromosome‐scale genome sequence assembly. EBH wrote the manuscript with the help of MvK.

## Disclaimer

The New Phytologist Foundation remains neutral with regard to jurisdictional claims in maps and in any institutional affiliations.

## Supporting information


**Fig. S1** Quantitative leaf characteristics of the leaf below the flag leaf in *H. erectifolium*, cultivated (Morex) and wild barley.
**Fig. S2** Principal component analysis of tissue‐specific expression profiles.
**Fig. S3** Putative centromere locations and pericentric sizes in *H. erectifolium*, Morex, and B1K‐04‐12.
**Fig. S4** *Copia* and *Gypsy* LTR retrotransposon insertions over time in chromosomes 2H and 7H.
**Fig. S5** Enriched biological pathways found in hierarchical phylogenetic orthologs.
**Fig. S6** Summary of expanded and contracted gene families found by CAFE5.
**Fig. S7** Significantly expanded gene families found in *H. erectifolium* related to desiccation tolerance.
**Fig. S8** Leaf relative water content and plant morphology during drydown and recovery.
**Fig. S9** Soil field capacity and fresh weight biomass during drydown and recovery.
**Fig. S10** Time‐course analysis of transcriptome changes over time in response to drydown.
**Fig. S11** Gene expression of expanded hierarchical phylogenetic orthologs gene families in response to drydown and recovery.
**Methods S1** Online methods.

**Table S1** Oxford Nanopore Technologies long read sequencing data.
**Table S2** 10x Genomics linked short read Illumina sequencing data.
**Table S3** Hi‐C chromosomal conformation capture Illumina sequencing data.
**Table S4** Bionano optical genome maps.
**Table S5** Genome assembly metrics, completeness and quality score. From draft assembly to final pseudomolecules.
**Table S6** Overview of PacBio IsoSeq full‐length capture transcript RNA sequencing of tissue‐time specific samples.
**Table S7** Gene expression in the 22 tissue and time‐specific samples, read counts (TPM) normalized per sample with IsoQuant.
**Table S8** Summary of gene annotation, combined from evidence‐based gene prediction and *ab initio* prediction, and lncRNA.
**Table S9** Transposable element annotation with EDTA in *H. erectifolium* acc. NGB6816, *Hv*. cv Morex, and *Hv. spontaneum* acc. B1K‐04‐12.
**Table S10** Total number of expanded or contracted hierarchical phylogenetic orthologs (HOG).
**Table S11** All significantly expanded or contracted hierarchical phylogenetic orthologs (HOG) in *H. erectifolium*.
**Table S12** Expanded gene families of desiccation tolerance genes *ELIP*, *DREB1C*, and *LEA*.
**Table S13** Tissue and time‐specific expression of the expanded gene families: *ELIP*, *DREB1C*, and *LEA* genes in the 22 IsoSeq samples.
**Table S14** Transcriptome sequencing with Illumina PE150 of 64 leaf samples from *H. erectifolium* and Morex during the drydown experiment.
**Table S15** RNAseq mapping rates of each sample to their respective genome.
**Table S16** Total number of significantly expressed genes found in *H. erectifolium* and Morex during the drydown experiment.
**Table S17** All expressed genes detected in *H. erectifolium* during the drydown experiment.
**Table S18** All expressed genes detected in Morex during the drydown experiment.
**Table S19** Cross‐species comparison of differentially expressed single‐copy orthologs between *H. erectifolium* and Morex.
Please note: Wiley is not responsible for the content or functionality of any Supporting Information supplied by the authors. Any queries (other than missing material) should be directed to the *New Phytologist* Central Office.

## Data Availability

The raw sequencing data were deposited at European Nucleotide Archive (ENA). The Oxford Nanopore, 10× Genomics Linked‐Reads and drydown RNAseq raw data are under accession ID PRJEB104866. Chromosomal conformation capture (Hi‐C) sequence data are available under accession no. SAMEA118868558 (PRJEB94868), and the PacBio IsoSeq sequences under accession no. SAMEA118907054 (PRJEB94869). The data that support the findings of this study are openly available at DataPLANT (Weil *et al*., 2023), our FAIR data publication can be found under doi: https://doi.org/10.60534/qv5j9‐srm13.
