# Supplementary material for: A chromosome‐scale genome assembly of Hordeum erectifolium : genomic, transcriptomic and anatomical adaptations to drought in a wild barley relative
Source: New Phytol. 2026 Mar 24;250(4):2652–69. doi: 10.1111/nph.71091 (PMC13103428; doi:10.1111/nph.71091)
Supplement: Supplementary file 1 — Fig. S1 Quantitative leaf characteristics of the leaf below the flag leaf in H. erectifolium, cultivated (Morex) and wild barley. Fig. S2 Principal component analysis of tissue‐specific expression profiles. Fig. S3 Putative centromere locations and pericentric sizes in H. erectifolium, Morex, and B1K‐04‐12. Fig. S4 Copia and Gypsy LTR retrotransposon insertions over time in chromosomes 2H and 7H. Fig. S5 Enriched biological pathways found in hierarchical phylogenetic orthologs. Fig. S6 Summary of expanded and contracted gene families found by CAFE5. Fig. S7 Significantly expanded gene families found in H. erectifolium related to desiccation tolerance. Fig. S8 Leaf relative water content and plant morphology during drydown and recovery. Fig. S9 Soil field capacity and fresh weight biomass during drydown and recovery. Fig. S10 Time‐course analysis of transcriptome changes over time in response to drydown. Fig. S11 Gene expression of expanded hierarchical phylogenetic orthologs gene families in response to drydown and recovery. Methods S1 Online methods. [file NPH-250-2652-s001.pdf]

New Phytologist Supporting Information

Article title: A chromosome-scale genome assembly of *Hordeum erectifolium*: genomic, transcriptomic and anatomical adaptations to drought in a wild barley relative

Authors: Haraldsson, Einar Baldvin; Anokye, Michael; Rütjes, Thea; Toegelová, Helena; Tulpová, Zuzana; Šimková, Hana; Feng, Jia-Wu; Mascher, Martin; von Korff, Maria

Article acceptance date: 12 February 2026

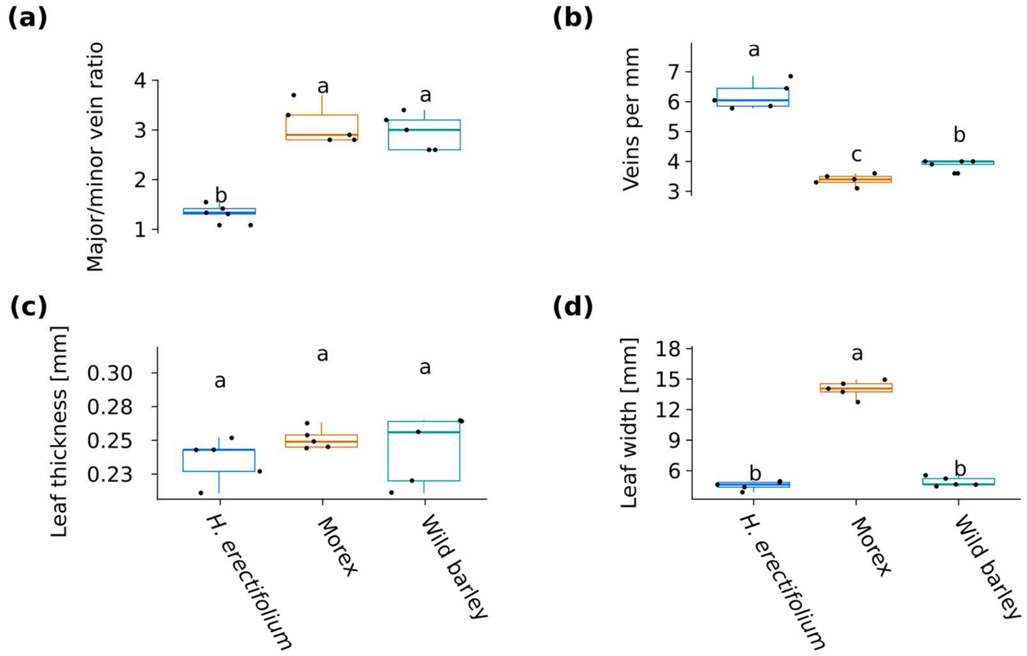

Fig. S1: Quantitative leaf characteristics of the leaf below the flag leaf (LBF) in *H. erectifolium*, cultivated (Morex) and wild barley.

(a) Minor to major vein ratio and **(b)** number of veins per mm in the LBF. **(c,d)** LBF thickness and width in mm. Boxplots show the distribution of values (black dots), center line shows the median, lower and upper hinges denote the first and third quartile, respectively, and the upper and lower whiskers extend no further than 1.5 times the inter-quartile range of their respective hinge. Number of samples, $n = 5$, different letters indicate significantly differing groups, ANOVA with post-hoc Tukey HSD, $p < 0.05$.

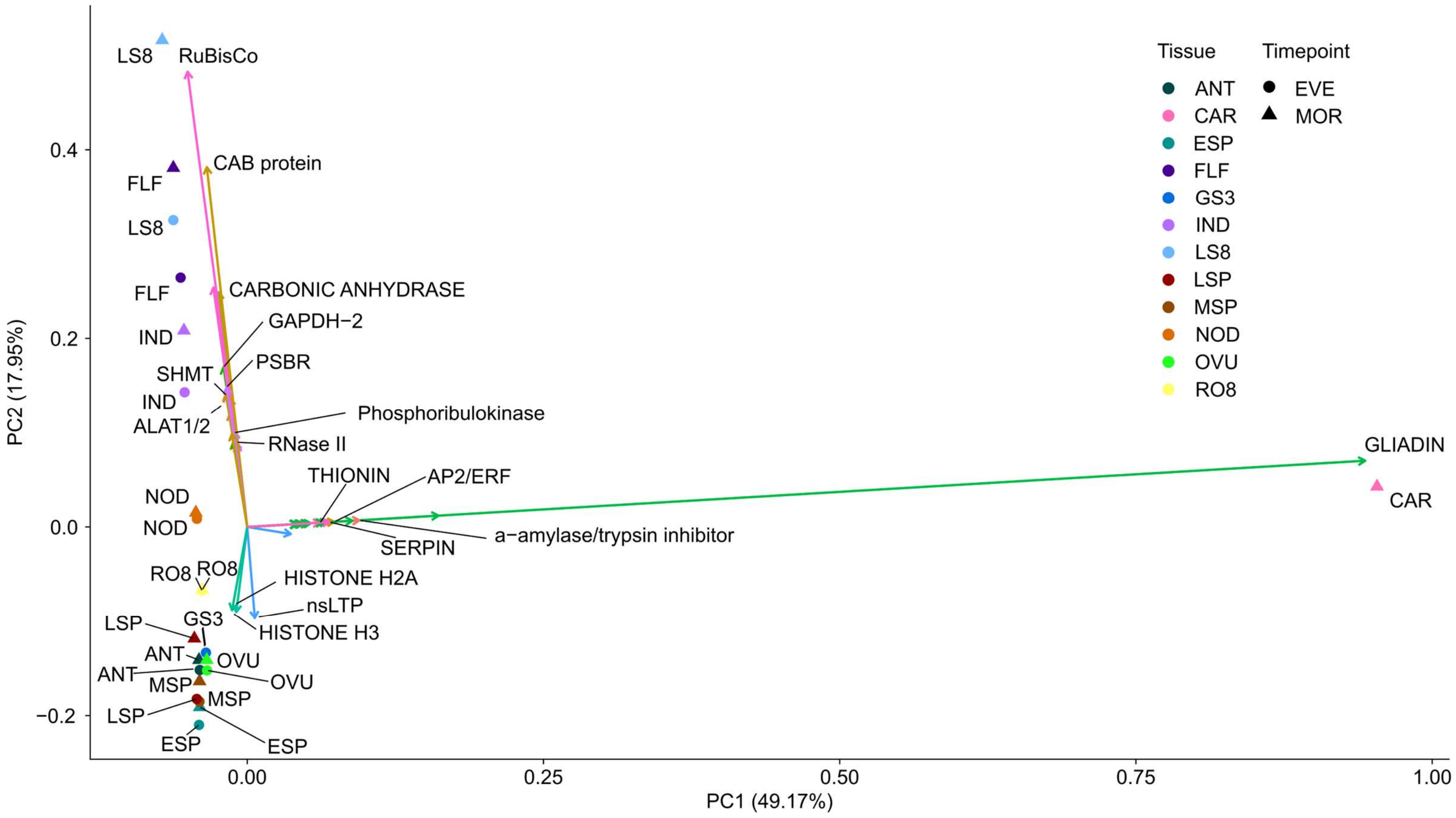

Fig. S2: Principal component analysis (PCA) of tissue-specific expression profiles.

PCA clustering of transcript abundance in 22 tissue-time specific samples and the trajectory of the top 20 highest loading genes for PC1 and PC2. Twelve individual tissues of which ten were sampled both in the morning (MOR, ZT 1-3) and evening (EVE, ZT 13-15). Three main trajectories; lower left: spike development and reproductive tissues, upper left: vegetative tissues, influenced by photosynthesis and carbon metabolism-related genes, right: CAR_MOR (caryopsis, 10 days post anthesis) explaining 49 % of the variance on PC1 due to high expression from multiple *GLIADIN* genes. Abbreviations: flag leaf (FLF), three-day-old germinating seeds (GS3), third internode (IND), fourth node (NOD), whole shoots, (LS8, 8 days post germination), whole roots (RO8, 8 days post germination), ovules (OVU), anthers (ANT), caryopses (CAR), developing shoot apical meristems: ESP (W3.0-4.5), MSP (W5.0-6.5), LSP (W7.0-8.0) (Waddington et al., 1987).

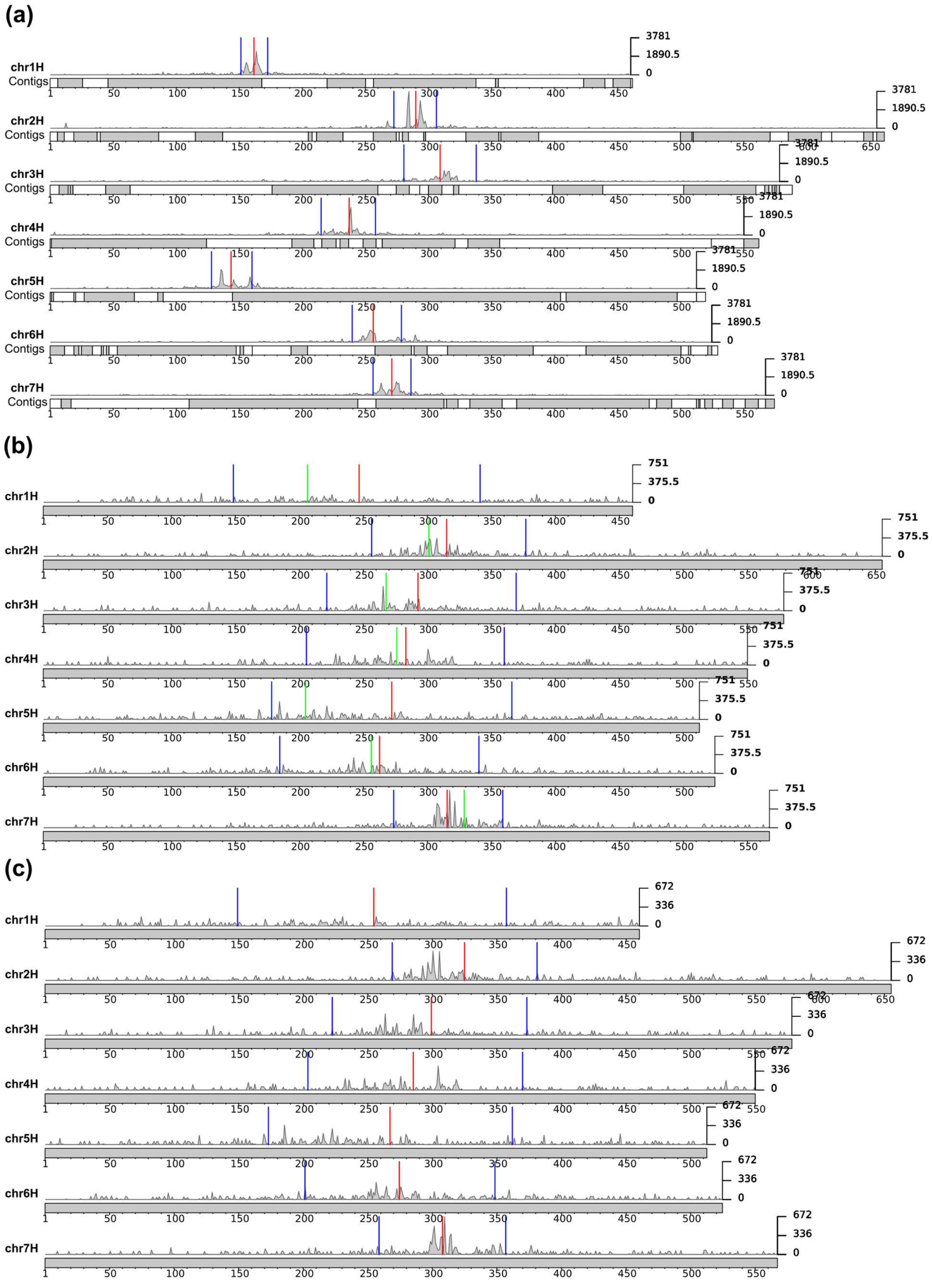

Fig. S3: Putative centromere locations and pericentric sizes in *H. erectifolium*, Morex, and B1K-04-12.

(a) *H. erectifolium* showing putative centromeric locations and contig boundaries in the assembled pseudomolecules (chromosomes). Putative centromeric locations in **(b)** Morex and **(c)** B1K-04-12. Density of aligned *CRM* LTR retrotransposon sequences in 1 Mb windows across chromosomes. The red line indicates the predicted midpoint of the *CRM* LTR clade density at the putative centromere midpoint, blue lines show the 50 % confidence limits, here used as an estimate of centromere boundaries and the green lines indicate previously published centromere locations for Morex (Mascher et al., 2021).

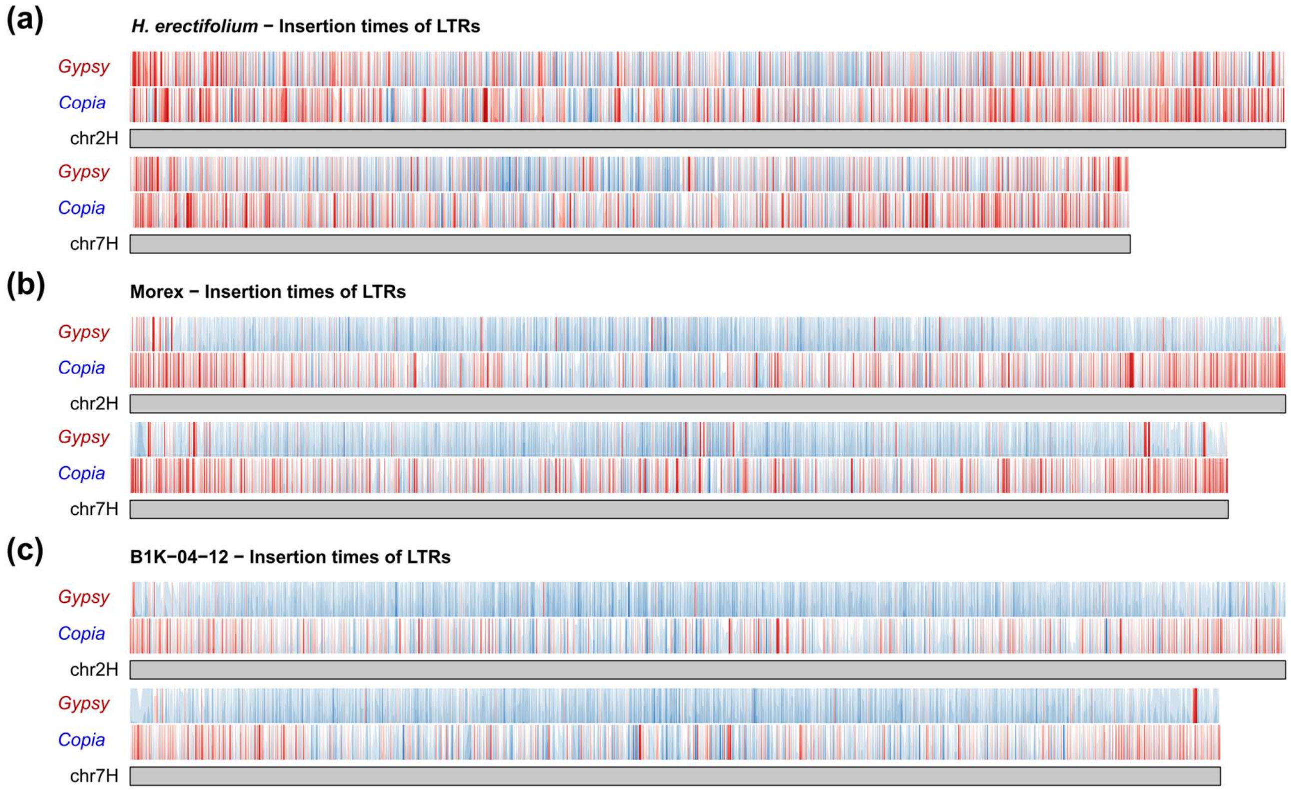

Fig. S4: *Copia* and *Gypsy* LTR retrotransposon insertions over time in chromosomes 2H and 7H

LTR retrotransposon insertion times were normalized and scaled to show the relative time of the most recent insertions of *Gypsy* (red) and *Copia* (blue) across two example chromosomes, 2H and 7H. There were greater frequencies of recent *Copia* insertions at the chromosomal ends of all three genomes. *H. erectifolium* had *Gypsy* insertions that are spread across the entire chromosomes, but in Morex and B1K-04-12, there were few insertions and mainly towards the far ends. Normalization of insertion times for superfamilies *Copia* and *Gypsy* were scaled to the oldest insertion found at ~6.4 Mya and a heatmap corresponding to the age of insertion along the chromosomes, <0.6 Mya (red) and >0.6 Mya (blue).

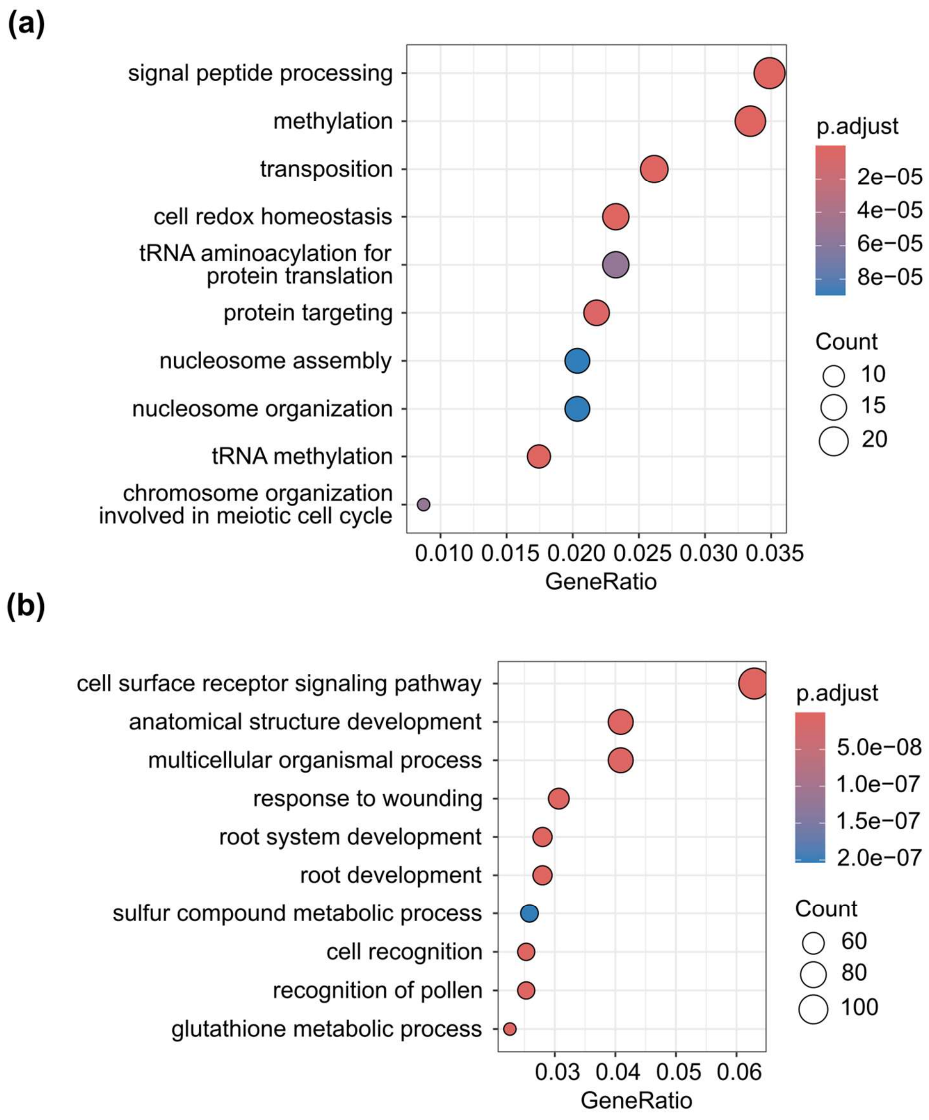

Fig. S5: Enriched biological pathways found in hierarchical phylogenetic orthologs

(a) Enriched biological pathway terms in hierarchical phylogenetic orthologs (HOG) that were unique to *H. erectifolium*. **(b)** Enriched biological pathways of significantly expanded or contracted HOGs in *H. erectifolium* revealed by CAFE5 analysis.

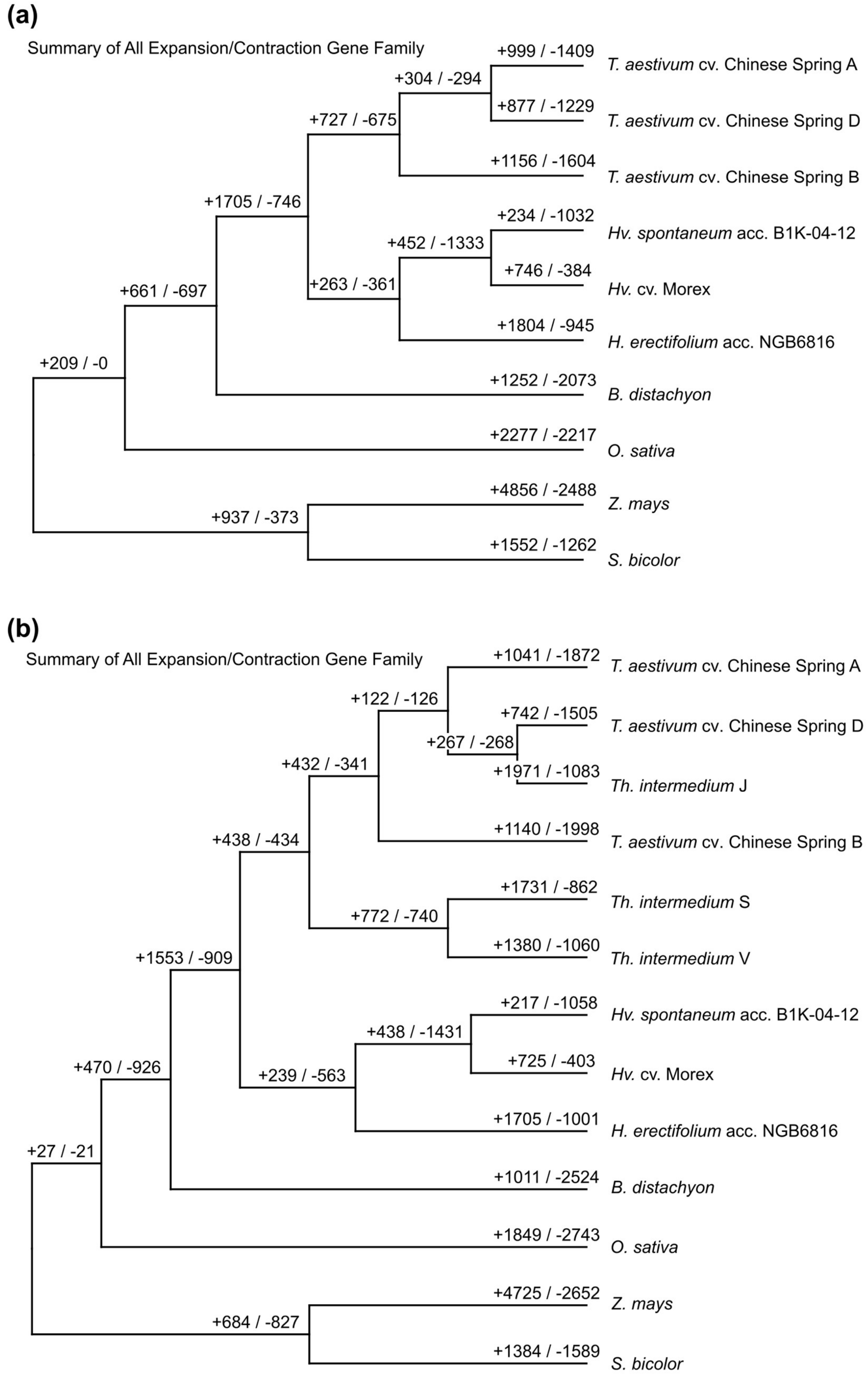

Fig. S6: Summary of expanded and contracted gene families found by CAFE5.

Total number of expanded and contracted hierarchical phylogenetic orthologs (HOG), found by CAFE5 analysis. **(a)** without *Th. intermedium* and **(b)** with. Numbers indicate the number of expanded (+) or contracted (-) at each node.

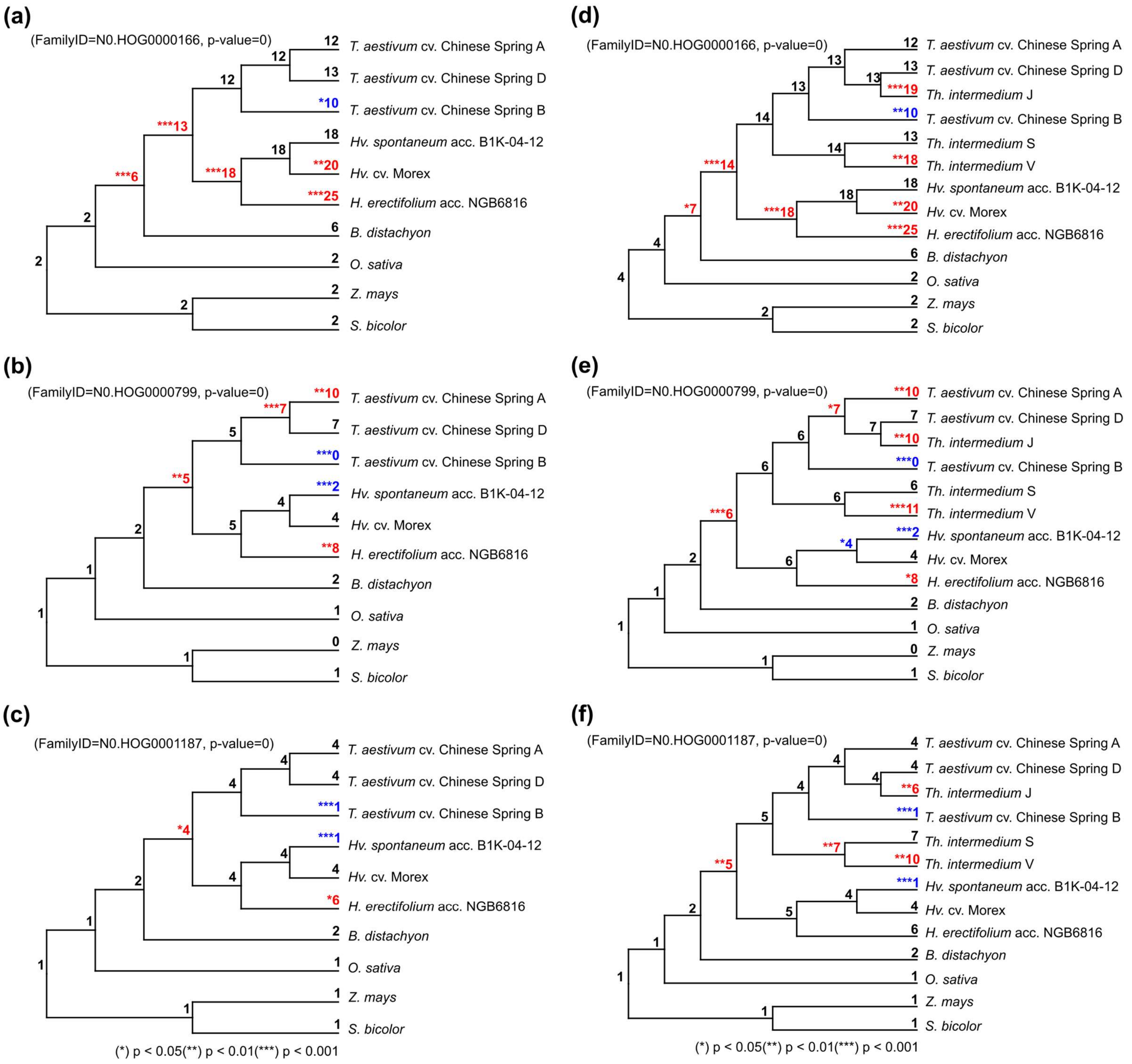

Fig. S7: Significantly expanded gene families found in *H. erectifolium* related to dessication tolerance.

Significantly expanded hierarchical phylogenetic orthologs (HOG), on the branch of *H. erectifolium* **(a, d)** *EARLY LIGHT-INDUCED PROTEINS* (*ELIP*), N0.HOG0000166, **(b, e)** *LATE EMBRYOGENESIS ABUNDANT PROTEIN 6-RELATED* (LEA PROTEIN 6-RELATED), N0.HOG0000799, **(c, f)** *DEHYDRATION-RESPONSIVE ELEMENT-BINDING PROTEIN 1C* (*DREB1C*), N0.HOG0001187. **(a-c)** without *Th. intermedium* and **(d-f)** with. Numbers indicated number of genes, red marks significantly expanded and blue significantly contracted, *, p < 0.05, **, p < 0.01, ***, p < 0.001.

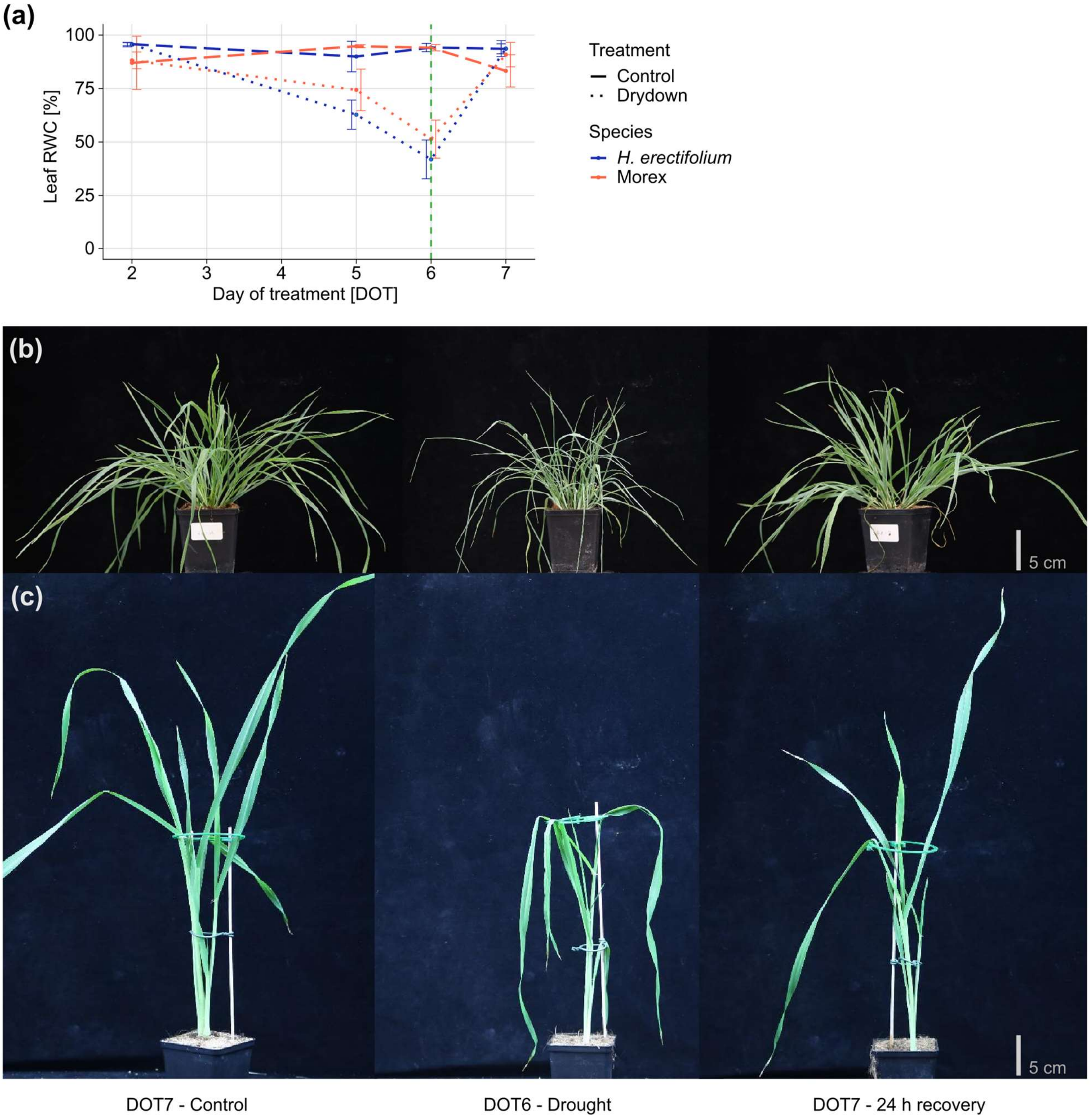

Fig. S8: Leaf relative water content and plant morphology during drydown and recovery.

(a) Leaf relative water content (RWC) measured at each sampling time point, *H. erectifolium* (blue) and Morex (orange), starting at ~100 % and ending at ~50 % on DOT6, and ~100 % 24 hours after rewatering, on DOT7. Dotted lines – drydown , dashed line – control, n = 4, error bars are standard deviation, two plants were pooled at each timepoint and treatment. **(b, c)** Morphology of *H. erectifolium* and Morex during drydown, with control on DOT7 (left), drydown on DOT6 (middle) and DOT7 recovery (right). Leaves of *H. erectifolium* rolled inward and maintained erectness (DOT6), while Morex leaves wilted and needed to be supported during treatment.

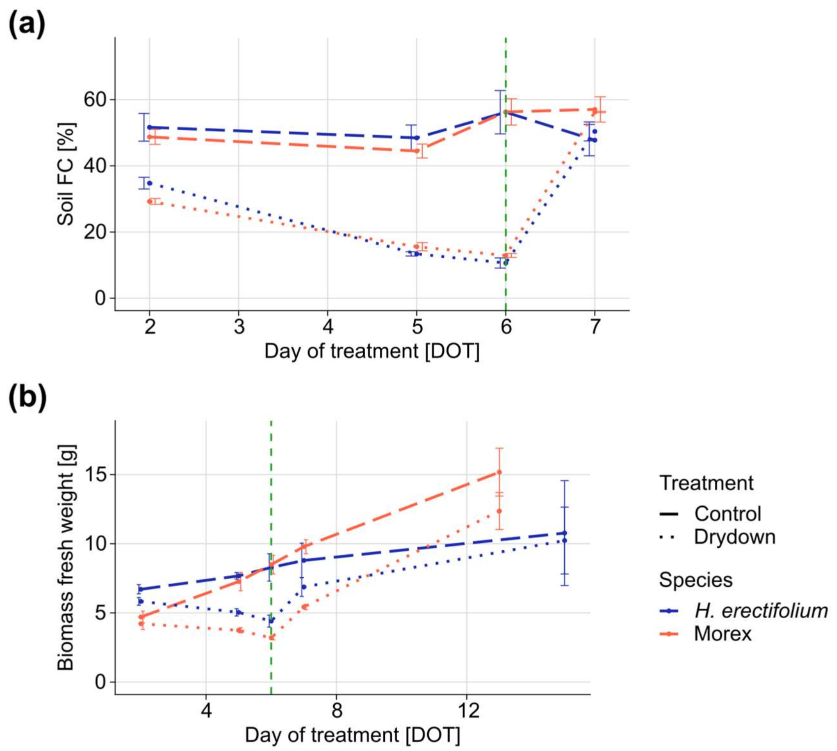

Fig. S9: Soil field capacity and fresh weight biomass during drydown and recovery.

(a) The control group was maintained at 50 % soil field capacity (FC), water was withheld for six days after adjusting soil FC to 50 % and rewatered to 50 % FC on the sixth day of treatment (DOT), vertical black dotted line. FC was measured at each sampling time point *H. erectifolium* (blue) and Morex (orange), drydown (dotted lines), control (dashed line), n = 4, error bars are standard deviation, two plants were pooled at each timepoint and treatment. **(b)** Progression of fresh weight biomass during and after recovery of drydown treatment at DOT15 and DOT13 for *H. erectifolium* and Morex, respectively.

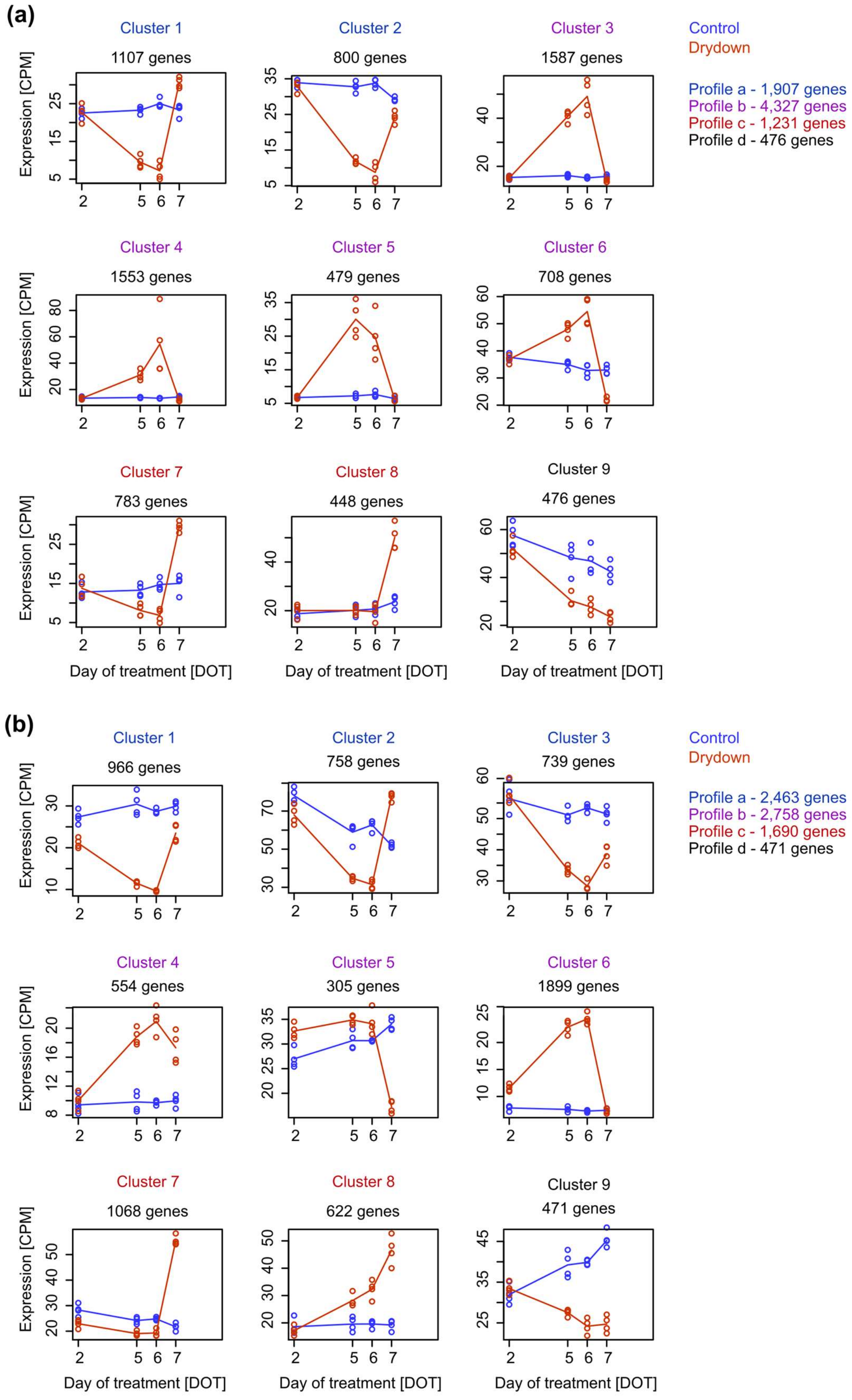

Fig. S10: Time-course analysis of transcriptome changes over time in response to drydown.

Differentially expressed genes (DEG) over time during drydown treatment, transcriptome analysis with maSigPro and DEGs clustered into nine clusters. **(a)** *H. erectifolium* (7,941 DEGs total), expression pattern clusters were grouped into a) 1, 2 (blue), 1,907 DEGs; b) 3, 4, 5, 6 (purple), 4,327 DEGs; c) 7, 8 (red), 1,231 DEGs; d) 9 (black), 476 DEGs. **(b)** Morex (7,382 DEGs total), expression pattern clusters were grouped into a) 1, 2, 3 (blue), 2,463 DEGs; b) 4, 5, 6 (purple), 2,758 DEGs; c) 7, 8 (red), 1,690 DEGs; d) 9 (black), 471 DEGs. Significant DEGs from time-course analysis in maSigPro were selected with an R^2 fit of >= 0.7 and assigned to nine expression profile clusters with hclust with their normalized read counts. Control in blue and drydown as orange.

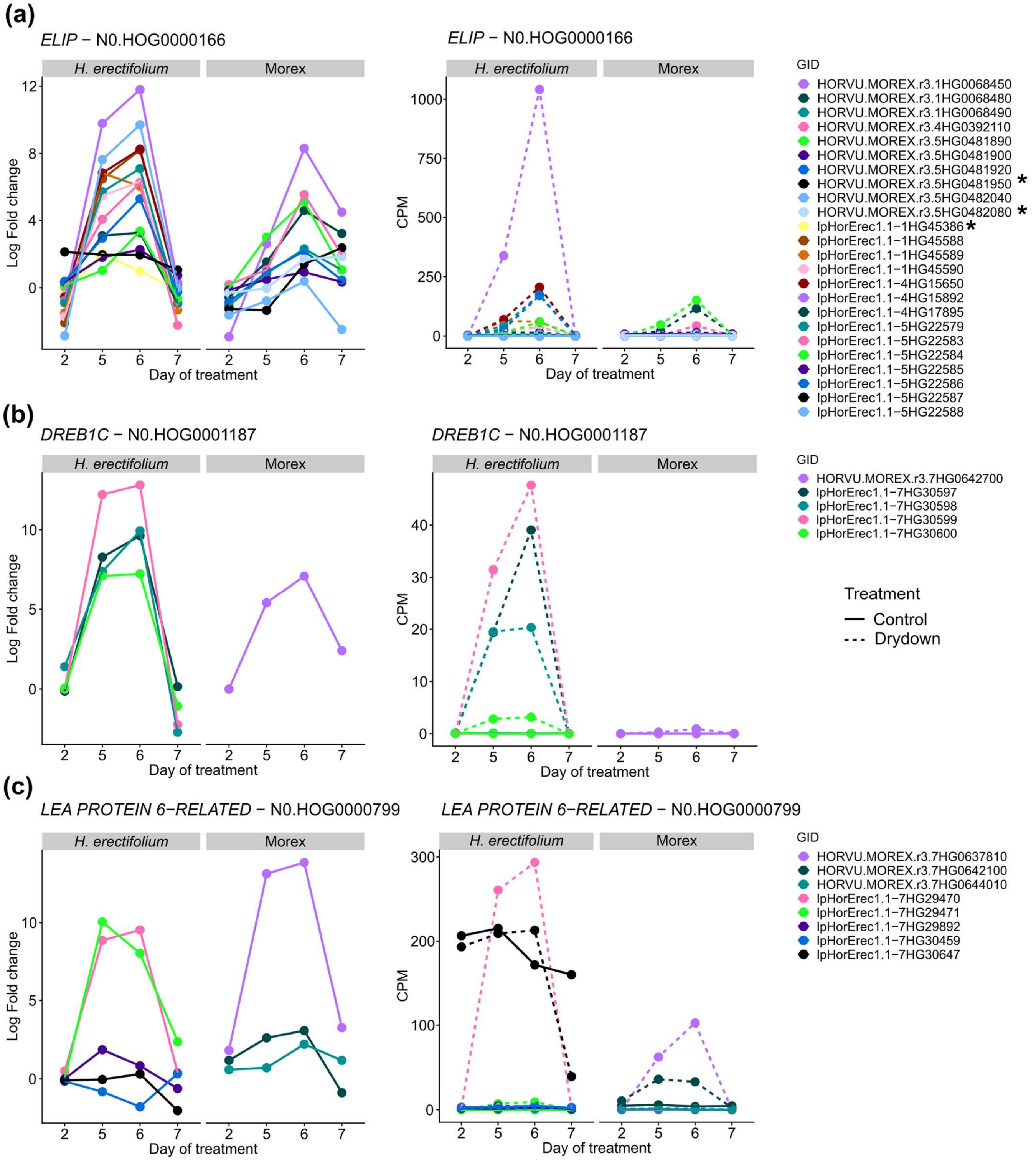

Fig. S11: Gene expression of expanded hierarchical phylogenetic orthologs gene families in response to drydown and recovery.

Expressed genes of expanded hierarchical phylogenetic orthologs (HOG) gene families; **(a)** *EARLY LIGHT-INDUCED PROTEINS* (*ELIP*) (N0.HOG0000166), **(b)** *LATE EMBRYOGENESIS ABUNDANT PROTEIN 6-RELATED* (*LEA PROTEIN 6-RELATED*) (N0.HOG0000799), and **(c)** *DEHYDRATION-RESPONSIVE ELEMENT-BINDING PROTEIN 1C* (*DREB1C*) (N0.HOG0001187), which expanded on the phylogenetic branch of *H. erectifolium*. Graphs show both the log fold change (left) and counts per million (CPM) (right) values for control and drydown. All genes, except those marked with an asterisk, were differentially regulated in response to drydown or recovery.

Methods S1

Plant material and growth conditions

For all experiments, we used single descent propagated seeds of *Hordeum erectifolium* Bothmer, N.Jacobsen & R.B.Jørg. acc. NGB6816 (Nordic Genetic Resource Center, Sweden), and *Hordeum vulgare* var. *spontaneum* (K.Koch) Körn. acc. B1K-04-12 from the Barley1K collection (Hübner *et al.*, 2009), *Hv.* var. *spontaneum* (K.Koch) Körn. acc. HID-4 (Liller *et al.*, 2017), and *H. vulgare* L. cv. Morex. *H. erectifolium* and Morex were used in all experiments, but B1K-04-12 was used for leaf anatomical phenotyping, and HID-4 for specific leaf area (SLA) and elemental carbon and nitrogen measurements.

Unless specified otherwise, plants were consistently grown under the following conditions. Seeds were sown in a mixture of 93% (v/v) Einheitserde ED73 (Einheitserdewerke Werkverband e.V., Sinntal-Altengronau, Germany), 6.6% (v/v) sand, and 0.4% (v/v) Osmocote exact standard 3-4M (Scotts Company LLC). Stratified at 4 °C before being placed in a growth chamber with long day conditions (16h light, 8h dark, at 20°C day/16°C night, 60% relative humidity) for germination. Ten days after germination, they were then vernalized for 8 weeks at 4 °C under short-day conditions (8 h light, 16 h dark, at 4 °C day/4 °C night), before being transferred back to long-day conditions.

Plants used for specific leaf area and elemental carbon and nitrogen measurements were cultivated as described above. After vernalization, they were repotted to 7.5 L pots and grown further in a common garden at the Botanical Garden (BG) of the Heinrich-Heine-Universität Düsseldorf (HHU), data collected were from three summer seasons of 2021 – 2023.

Leaf transverse cutting and staining

The flag leaf and the leaf below the flag leaf were collected, and 1 cm sections at the midpoint of the leaf were cut. The sample was cut transversely with a razor blade by hand, and sections were placed in a 1.5 mL tube with fixing solution of Ethanol:Acetic acid (6:1 v/v). The tubes were then placed on top of a rotary shaker for two hours at 20-50 RPM. After two hours, the fixing solution was removed and 85 % (v/v) ethanol was added and stored until microscopy. The fixed sections were washed three times with dH_2O and stained in 0.5 % (w/v) toluidine blue for 10 seconds. The staining solution was removed by washing three times with dH_2O, and sections were transferred to a slide for imaging. Images of leaf sections were obtained using a Nikon stereo microscope (Nikon SMZ18), Nikon DS-U3 controller unit, and a Nikon DS-Fi2 digital camera. Nikon NIS-Element's software was used for image acquisition (Nikon Corporation, Tokyo, Japan).

Growth conditions for the field experiment

Seeds were pre-germinated in 96-well trays manually filled with a freshly prepared soil mixture consisting of 99.6% (v/v) Mini tray soil (MIM800, Balster Einheitserdewerk, Fröndenberg, Germany) and 0.4% (v/v) Osmocote Exact Standard 3-4M (Scotts Company LLC). A single grain was sown 1cm deep in each well and bottom watered with 2 liters of water per tray. Trays were then placed in a controlled-environment growth cabinet, where grains were cold stratified for 14 days at 4 °C in the dark to promote uniform and synchronous germination. After stratification, the trays were transferred to long-day (LD) conditions in a controlled phytotron. Temperature was set to 22: 18°C, day: night, and a 16 h photoperiod. The seedling emergence was scored and varied 5-10 days after transfer to LD growing conditions. Once seedlings developed two to three leaves, they were moved to short-day (SD) conditions (8h light/ 16h dark) and 4°C (day and night). Seedlings were thus vernalized for 12-14 weeks before transplanting to outdoor conditions.

The experiments were conducted under outdoor conditions at the Botanical Garden (BG) of Heinrich-Heine-Universität (HHU), Düsseldorf, Germany, during the summer seasons of 2021, 2022, and 2023 (March/April to August each year). In each experiment, individual seedlings of H. erectifolium, Morex and wild barley were repotted into 7.5 L pots (25.9 cm x 20.6 cm), manually filled with 7 L of 93 % (v/v) Einheitserde 'ED73' peat soil (Einheitserde Werkverband e.V., Sinntal Altengronau, Germany), 6.6 % (v/v) sand, and 0.4 % (v/v) Osmocote exact standard 3-4M (Scotts Company LLC). The seedlings were arranged in a randomized complete block design (RIBD) of four blocks. Each accession was represented by 4 plants per block, resulting in a total of 16 replicate plants. These plants were used for measurements of leaf morphology and nitrogen and carbon content as described below. During the growth period, water was manually provided 2-3 times a week, except on rainy days, until the experiment concluded.

No additional fertilization, disease, or pest management practices were applied for all seasons. Air temperature for the experiment locations was monitored on-site with a Spectrum WatchDog 2900ET Weather Station (Spectrum Technologies, Inc., Fairborn, USA).

Specific leaf area measurement

The specific leaf area (SLA) was measured by scanning the area (cm^2) of a main culm flag leaf. The leaf area (leaves rehydrated for 24 h in 50 mL tubes) was determined using Petiole (v. 4.0.2, Petiole LTD, U.S.A.) mobile application (petioleapp.com) installed on Galaxy Tab Active3 (Samsung, South Korea). All leaf area measurements were performed after calibration with PETIOLE Calibration Pad N°5 (https://petioleapp.com/pads/Petiole-calibration_pad_5.pdf, retrieved on 05.05.2021). Afterwards, the leaf was dried for 48 h at 65 °C, and the dry weight was measured. The leaf area and dry weight measurements were used to calculate the SLA as follows: SLA = leaf area (cm^2) / dry weight (mg).

Sample preparation and measurement of elemental carbon and nitrogen in leaves

Elemental carbon (C) and nitrogen (N) measurements were performed using flag leaf samples collected from the three oldest reproductive tillers per plant at the grain-filling stage, during two seasons, 2021 and 2022. Flag leaves were cut into paper bags from four biological replicates for each genotype. Approximately 0.02 g of the harvested leaves was shredded into a 2 mL reaction tube with 2 (3 mm) steel beads and dried at 65 °C for 72 h. Following drying, leaves were ground to a fine powder in Qiagen TissueLyser II (QIAGEN GmbH, Hilden, Germany). Accurately approximately 2 mg of homogenized leaf powder was weighed (XP6/52, Mettler Toledo, Switzerland) per sample. C and N concentration in the leaf was determined using the Isoprime 100 isotope ratio mass spectrometer coupled to an isotope cube elemental analyzer (Elementar, Hanau, Germany), adapting procedures previously described (Coplen *et al.*, 2006; Gowik *et al.*, 2011). All C and N measurements were done at the CEPLAS Metabolomics and Metabolism Laboratory (CMML, Heinrich-Heine University Dusseldorf, Germany).

***H. erectifolium* high molecular weight DNA isolation**

A single plant of *H. erectifolium* acc. NGB6816 was grown under control conditions and used for extracting high molecular weight (HMW) DNA. The protocol was based on the Oxford Nanopore Technologies (ONT) HMW DNA extraction protocol (Oxford Nanopore Technologies, 2021). One gram of fresh young leaves was ground in liquid nitrogen to a fine powder with a pestle and mortar. HMW DNA was extracted in 20 mL of a modified Carlson lysis buffer (100 mM Tris-HCl, pH 9.5, 2 % CTAB, 1.4 M NaCl, 2 % PVP-40, 20 mM EDTA, 3mM EGTA, 0.25 % beta-mercaptoethanol, 0.25 mg Rnase A), followed by purification using a Qiagen Genomic-tip 100/G (Qiagen, Cat. No: 10243). Purified HMW DNA was eluted from the column in 500 µL EB (10 mM Tris-HCl, pH 8.0). Concentrations (A260/A230) and purity (A260/A280) were measured on a Nanophotometer NP80 (IMPLEN, Germany).

HMW DNA sequencing

There were 24 ONT libraries sequenced on GridION R9.4.1 (FLO-MIN106) flowcells at the Max Planck-Genome-Centre Cologne (Cologne, Germany), of those, 21 ONT libraries were prepared with library kit SQK-LSK109 and three with SQK-RAD004. Further three libraries were sequenced on PromethION R9.4.1 (FLO-PRO002) flowcells prepared with library kit SQK-LSK110, and were sequenced at the Genomics & Transcriptomics Laboratory, HHU (Düsseldorf, Germany). Furthermore, we sent HMW DNA to Novogene (Novogene (UK) Company Limited, Cambridge, UK), for 10x Genomics Linked-Reads sequencing.

Hi-C sequencing

In situ Hi-C libraries were prepared from young seedlings (seeds from the same *H. erectifolium* plant from which HMW DNA was sampled) at IPK Gatersleben (Germany) according to the previously

published protocol, using *Dpn*II for the digestion of crosslinked chromatin. Sequencing and Hi-C raw data processing were performed as described before (Himmelbach *et al.*, 2018a; Himmelbach *et al.*, 2018b).

Tissue-time specific sampling and RNA extraction

All RNA plant tissue samples were collected from the same plant as was previously harvested for DNA; seedlings and germinating seeds were from seeds of that same plant. The seeds were cleaned, and only the main central grain was selected; thereafter, the seeds were soaked in water overnight at RT in a 2 mL tube. Water was removed, and the seeds were sterilized with 1 mL of 0.5 % (v/v) sodium hypochlorite solution, the seeds were incubated for 20 minutes, and the tube was inverted every four minutes. The sodium hypochlorite solution was removed, and the seeds were washed four times with dH_2O, before being plated on moist filter paper in a plastic Petri dish. Petrie dishes were sealed with Parafilm, covered in aluminium foil and placed in a 4 °C incubator for five days for stratification; thereafter, they were placed in a growth chamber under control conditions. Germinating seeds were then transplanted into 0.7 % (w/v) Phyto agar (Prod. No: P1003.1000, Duchefa Biochemie Haarlem, The Netherlands). We sampled a total of 12 tissues at two time points, Zeitgeber (ZT), morning (MOR, ZT 1-3) and evening (EVE, ZT 13-15); whole shoot (LS8) and whole root 8 (RO8), 8 days post germination, anthers (ANT) and ovules (OVU) at W10, three developmental pools of spikes by their Waddington stage (W3.0-4.5, ESP; W5.0-6.5, MSP; W7.0-8.0, LSP) (Waddington *et al.*, 1983), third internode (INT), fourth node (NOD, flag leaf (FLF), caryopses (CAR) 10 days post anthesis, three-day old germinating seed (GS3). CAR and GS3 were only sampled at one time point; in total, 22 tissue samples were collected. Total RNA was extracted with Qiagen RNeasy Plant Mini Kit (Qiagen, Hilden, Germany, Cat. No. / ID: 74904), with the addition of 0.2 % (v/v) beta-mercaptoethanol. Total RNA concentrations (A260/A230) and purity (A260/A280) were measured using a Nanophotometer NP80 (IMPLEN, Germany) and integrity on a 1 % agarose gel. PacBio (Pacific Biosciences of California, Inc., USA) IsoSeq sequencing was performed at the Genomics & Transcriptomics Laboratory, HHU Düsseldorf (Germany).

PacBio IsoSeq sequencing

The RNA of the 22 tissues was assessed using Qubit RNA HS Assay (Thermo Fisher Scientific Massachusetts, U.S.A.) and NanoDrop (Thermo Fisher Scientific, Massachusetts, U.S.A.) to check the concentration and purity, while the FragmentAnalyzer (DNF-471; Agilent Technologies, California, U.S.A.) was used to check the RNA integrity, RIN >= 8, before library preparation. Subsequently, the intact poly(A) RNA was captured and purified using the NEBNext Poly(A) mRNA Magnetic Isolation Module (New England BioLabs, Massachusetts, U.S.A.) following the manufacturer's instructions. Afterwards, the cDNA was generated according to the TeloPrime Kit Version 2 (Lexogen, Vienna,

Austria). The optimal number of cycles was determined by qPCR (QuantStudio 3, Thermo Fisher Scientific, Massachusetts, U.S.A.) with 0.1x SYBR Green (Merck Millipore, Darmstadt, Germany), TeloPrime kit chemistry and 10 % of the cDNA as input material. Based on the qPCR, the number of cycles was adjusted to yield the appropriate amount of amplified cDNA using barcoded Teloprime primer within the Endpoint-PCR. The quality of the mass-amplified full-length cDNA was assessed according to the manufacturer's instructions and then column-purified using the TeloPrime Kit Version 2 (Lexogen, Vienna, Austria). The samples were pooled in equal molar amounts, followed by the IsoSeq PacBio library preparation, using the IsoSeq Express Template Preparation for Sequel and Sequel II Systems (v.02, 2019) (Pacific Biosciences, California, U.S.A.) starting with the DNA damage repair step. Subsequently, the quality (FragmentAnalyzer, Agilent Technologies, California, U.S.A.) and quantity (Qubit, Thermo Fisher Scientific, Massachusetts, U.S.A.) was determined. The long-read sequencing was executed on a Sequel II system with two SMRT cells (8M), the Sequel II Binding kit 2.1, and the Sequel II Sequencing kit 2.0 (Pacific Biosciences, California, U.S.A.). The first pool, 12 samples, was sequenced on one SMRT Cell, loaded with 130 pM and an acquisition time of 30 h, while the second pool, 10 samples, was sequenced with 120 pM and a movie time of 24 h. For both SMRT Cells a 2 h immobilization step and a 2 h of pre-extension step with diffusion loading were performed before acquisition. The first raw subread dataset was processed with SMRT Link v9.0, while the second dataset was processed with v10.1 (Pacific Biosciences, California, U.S.A.) to generate the circular consensus sequences (CCS) reads, internally using ccs v.4.0.1. cDNA barcodes introduced with the TeloPrime Kit Version 2 (Lexogen, Vienna, Austria) were trimmed via lima (v 2.1.0).

Genome size estimation

We estimated the genome size, ploidy level, and heterozygosity by *k*-mer frequency analysis using the 10X Genomics Linked-Reads (Illumina short-reads data). The raw 10X Genomics Linked-Reads data were processed with Long Ranger (v. 2.2.2) (10x Genomics, 2024) "longranger basic" with default parameters, generating paired-end Illumina PE150 short-reads data with Linked-Reads information in headers, hereafter referred to as short-reads. We used Jellyfish (v. 2.3.0) to count the 21-mers in the short-read data following calculation with findGSE (v. 1.94), (Marçais & Kingsford, 2011; Sun *et al.*, 2018). Parameters used were; Jellyfish: "jellyfish count *FASTQ* -C -m 21 -s 20G -o 21mer ; jellyfish histo -h 3000000 -o 21mer.histo", and findGSE: "findGSE(histo="21mer.histo", sizek=21) in R (v. 4.4.3).

ONT sequencing data processing and assembly

The raw ONT sequencing, fast5 files, data were basecalled with Guppy (v. 5.0.7) (Oxford Nanopore Technologies, 2024) using the Super Accuracy Model with the appropriate configuration, read quality filter of ≥ Q7, (Table S1), file for each flow cell and library kit combination (FLO-MIN106: dna_r9.4.1_450bps_sup.cfg or FLO-PRO002: dna_r9.4.1_450bps_sup_prom.cfg): "guppy_basecaller -

c *CONFIG* -min_qscore 7 -device cuda:0 -recursive -calib_detect". Read quality and length distribution were visualized with NanoPlot (v. 1.32.1): "-fastq -loglength -N50" (De Coster & Rademakers, 2023). Basecalled ONT long-read data were further processed with porechop (v. 0.2.4): "-check_reads 1000 -discard_middle" and then assembled with Flye (v. 2.9b1774): "-g 4.4g -m 10000 -nano-hq -extra-params max_bubble_length=300000" (Wick *et al.*, 2017; Kolmogorov *et al.*, 2019).

Assembly polishing with ONT long-read and 10X Genomics Linked-Reads short-reads

As part of the Flye assembly pipeline, Flye performed the first round of assembly polishing using ONT long-reads and was then sequentially polished, with the next long-read polishing steps performed as recommended by ONT. First, all ONT long-reads were mapped to the assembled contigs with minimap2 (v. 2.22-r1110-dirty): "-ax map-ont -2 -I 10G -K 50G -secondary=no" (Li, 2018), and then polished with Racon (v. 1.4.21): "-m 8 -x -6 -g -8 -w 500", ONT reads were re-mapped to the output and subsequently polished with Medaka (v. 1.4.3) (Oxford Nanopore Technologies, 2024): "-model r941_prom_sup_g507" (Vaser *et al.*, 2017). Finally, we mapped the pre-processed short-read data to the long-read polished assembly with BWA-MEM2 (v. 2.2.1) using default settings (Vasimuddin *et al.*, 2019). The BAM file was curated with Samtools (v. 1.15): "samtools view - -Sb -F 256 | samtools sort -" and two rounds of short-read polishing were performed with Hapo-G (v. 1.1) "-genome *ASM* -b *BAM* -o *OUT* -u", with the short-reads re-mapped to the output of first round of polishing (Aury & Istace, 2021; Danecek *et al.*, 2021).

Assembly correction and scaffolding with 10x Genomics Linked-Reads

After final polishing of the assembled contigs, we used the 10x Genomics Linked-Reads to correct and perform the first scaffolding of the polished contigs using Tigmint (v. 1.2.4)-ARCS (v. 1.2.2) pipeline with default parameters: "tigmint-make arcs " (Jackman *et al.*, 2018).

Optical genome mapping and hybrid scaffolding

A clone of the same *H. erectifolium* individual plant that was sequenced using ONT was used for the construction of the optical genome map (OGM). A total of 2.5 million nuclei, purified from young leaves by flow cytometry, were embedded in agarose miniplugs and treated with proteinase K, following the protocol described in Šimková *et al.* (2023). A total of 525 ng of HMW DNA was directly labeled at DLE-1 recognition sites using the standard Bionano Prep Direct Label and Stain (DLS) protocol (Bionano, San Diego, USA) and analyzed on the Bionano Saphyr platform. The resulting dataset, comprising 1.5 Tbp of single-molecule data with an N50 of 247 kb, provided approximately 341× coverage of the *H. erectifolium* genome (Table S4). This dataset was used to generate a *de novo* OGM assembly using Bionano Solve software (v. 3.6.1_11162020) with the standard configuration file "optArguments_nonhaplotype_noES_noCut_DLE1_saphyr.xml." To improve the contiguity of the ONT sequence assembly (*her_asm_allR9.4_final.fasta*), the automatic hybrid scaffold pipeline

integrated in Bionano Solve was run using the OGM assembly (Table S4). The default DLE-1 Hybrid Scaffold configuration file was applied with the “Resolve conflict” option for conflict resolution. Conflicts between the sequence assembly and the OGM were manually curated, and the pipeline was subsequently re-run using a modified *conflict_cut_status.txt* file.

Pseudomolecule construction

The hybrid-scaffolds were arranged to pseudomolecules with Hi-C chromosome conformation capture and the TRITEX pipeline, and orientation errors, pseudomolecule misassignments and chimeric contigs were manually curated by inspection of Hi-C contact matrices (Monat *et al.*, 2019). A unique genome assembly identifier, lpHorErec1.1, was retrieved and registered at ToLID (The Tree of Life Programme, 2025).

Assembly quality benchmarks and metrics

We estimated the assembly quality, completeness after each assembly polishing step and during scaffolding using the Merqury toolkit (Rhie *et al.*, 2020). A 21-mer database (db.meryl) was generated from the short-reads dataset with meryl (v. 1.4): “meryl k=21 count” and the Merqury pipeline was used to evaluate each step of the assembly, “merqury.sh db.meryl *ASM*” (Rhie *et al.*, 2020). Assembly metrics were collected using QUAST (v. 5.2.0), “quast.py -no-icarus -large” of the assemblies at each step of polishing and scaffolding (Mikheenko *et al.*, 2018). We used BUSCO (v. 5.4.7) with the Poales database with 4,896 genes, poales_odb10 (2020-08-05), to estimate genespace completeness of the assembly “-m genome” (Manni *et al.*, 2021).

PacBio IsoSeq processing and gene structural annotation

The PacBio IsoSeq sequencing data from the 22 samples were processed in bulk; we merged the individual samples together with Samtools merge (v. 1.16.1) and subsequently processed them with the isoseq3 (v. 3.8.2,) pipeline (Danecek *et al.*, 2021; 10x Genomics, 2024). First, we trimmed ploy-A tails and removed concatenated transcripts with isoseq3 “refine -require-polya -min-polya-length 12 teloprime.fasta”. The full-length non-concatenated (FLNC) reads were mapped to the genome with pbmm2 (v. 1.10.0) “align -preset ISOSEQ -sort -bam-index CSI” and redundant transcripts collapsed with isoseq3 “collapse -do-not-collapse-extra-5exons”. We predicted open reading frames (ORF) of the processed IsoSeq reads with TransDecoder (v. 5.7.0) “TransDecoder.LongOrfs -m 30 -S -complete_orfs_only” (Haas, 2023). Potential ORFs were further refined by aligning them with Diamond (v. 2.1.5) “-very-sensitive -outfmt 6 -evalue 1e-5 -max-target-seqs 1” to the UniRef90 2022_04 database and to Pfam v35 database with hmmscan (HMMER v. 3.3.2) “-domtblout” for the final ORF prediction selection, “TransDecoder.Predict -single_best_only” (HMMER development team, 2020; Blum *et al.*, 2021; Buchfink *et al.*, 2021; Haas, 2023; The UniProt Consortium *et al.*, 2023). Additional *ab initio* structural gene annotation was performed directly on the assembled genome with Helixer (v. 0.3.1) “-

lineage land_plant" model: land_plant_v0.3_a_0080.h5 (Holst *et al.*, 2025). Gene structural annotations predictions from both IsoSeq-TransDecoder and Helixer were evaluated for redundancy, with preference towards IsoSeq-TransDecoder if they differed only in UTR lengths but had identical internal exon structure. Final predictions were merged with AGAT (v. 1.2.0) (Dainat *et al.*, 2024). If there were multiple transcripts per gene which contained identical coding sequence (CDS) but differed only in their UTR lengths, we removed redundant transcripts by first clustering their CDS with CD-HIT (v. 4.8.1): "cd-hit -c 1 -n 5 -G 0 -d 0 -s 1 -aS 1 -AS 1 -AL 1" and selected a representative of isoforms sharing identical CDS structure (Fu *et al.*, 2012). A further representative isoform per gene was selected by aligning each protein-coding transcript against the UniRef90 (2023_02) protein database with Diamond (v. 2.1.8) "blastx -outfmt 6 qseqid sseqid pident length mismatch gapopen qstart qend sstart send evalue bitscore qlen slen qcovhsp scovhsp -ultra-sensitive -masking 0 -evalue 1e-05" (Buchfink *et al.*, 2021; The UniProt Consortium *et al.*, 2023). The isoform with the highest alignment score (based on bitscore, evalue, qcovhsp) was chosen as the representative isoform for that gene. For genes that did not have any hits in the reference database and the putative lncRNAs were selected based on the longest transcript as the representative.

Protein coding gene predictions were classified into high-(HC) or low-confidence (LC) categories. Predicted protein sequences were aligned with BLASTP to three manually curated databases; UniMag (reviewed (Swiss-Prot), Magnoliopsida (TaxID: 3398), UniProt release 2025_04), UniPoa (reviewed (Swiss-Prot) and Unreviewed (TrEMBL), Poaceae (TaxID: 4479), UniProt release 2025_04) (The UniProt Consortium *et al.*, 2023), and PTREP (TREP release 19) a database of hypothetical transposable element proteins (Schlagenhauf & Wicker, 2016). We considered it a best hit when a protein sequence had an e-value below 10-10 and query and subject coverage of 80% in one of the three databases. We classified a gene as HC when it had a best hit in UniMag, or in UniPoa but not in PTREP, otherwise it was classified as LC.

Functional gene annotation

Protein coding transcripts were functionally annotated and GO terms assigned with InterProScan-5.67-99.0 "-goterms -f tsv -dp", and additionally proteins were annotated with Mercator4 (v6.0) (Jones *et al.*, 2014; Bolger *et al.*, 2021).

lncRNA annotation

We put together a pipeline of nine tools to predict lncRNA for each transcript in the IsoSeq data; RNAsamba (v. 0.2.4), CPAT (v. 3.0.4), RNAplonc (v. 1.1), PlncPRO (v. 1.2.2), LncADeep (v. 1.0), CPPred (v. 2018-05-17), CPC2 (v. 0.1), CNCI (v. 2 Feb 28, 2014), PlncDB_V2.0 (Wang *et al.*, 2013; Sun *et al.*, 2013; Kang *et al.*, 2017; Singh *et al.*, 2017; Yang *et al.*, 2018; Negri *et al.*, 2019; Tong & Liu, 2019; Camargo *et al.*, 2020; Jin *et al.*, 2021). In short, if a transcript of a gene was predicted noncoding in seven out of nine predictors, subject cover less than 25 % and no hit of evalue <= 10e-5

in UniRef90 (2023_02) protein database after alignment with Diamond (v. 2.1.6) (Buchfink *et al.*, 2021), and had no domains predicted by InterProScan (v. 5.62-94.0) that gene it was assigned as Putative_lncRNA (Jones *et al.*, 2014; The UniProt Consortium *et al.*, 2023).

Genome comparative analysis

We compared the genomes of *H. erectifolium* acc. NGB6816 and two barley genomes, the barley reference MorexV3 (*H. vulgare* cv. Morex) and the wild barley genome *Hv. spontaneum* acc. B1K-04-12 (Jayakodi *et al.*, 2020, Mascher *et al.*, 2021). Hereafter, *H. erectifolium* acc. NGB6816 will be referred to as *H. erectifolium*; the two *H. vulgare* genomes will both be referred to as barley unless otherwise stated.

Transposable elements

Annotations of Transposable elements (TE) and repetitive regions in the assembled genome of *H. erectifolium* acc. NGB6816 and the two barley genomes were made with EDTA (v. 2.2.0) (Ou *et al.*, 2019), "EDTA.pl -anno 1 -cds libCDS.fa". To decrease the likelihood of genes being captured in the TE library, a curated library of CDSs, libCDS.fa, was generated for EDTA from a combined non-redundant library of high-confidence CDSs from *H. erectifolium* and MorexV3 gene annotations with CD-HIT (v. 4.8.1) "cd-hit -c 0.95 -n 5 -d 0" (Fu *et al.*, 2012). For the evaluation of long terminal repeat (LTR) retrotransposons clade clade-specific insertion times of intact LTR superfamilies Copia and Gypsy we additionally used TEsorter (v. 1.4.6) to further classify unknown LTR retrotransposon superfamilies found by EDTA with a higher sensitivity threshold, assigning them to either Gypsy or Copia using "-db rexdb-plant -rule 70-30-80" (Zhang *et al.*, 2022). Insertion times of intact LTRs were calculated by EDTA using the nucleotide substitution rate of $1.3x10^{-8}$ from *O. sativa*, as an indicator of their activation time (Ma and Bennetzen, 2004; Ou and Jiang, 2018). Intact LTR retrotransposons were then processed in R (v. 4.4), and LTR subclade insertion time frequencies were plotted with ggplot2 (Wickham, 2016; R Core Team, 2024). Normalization of insertion times for superfamilies Copia and Gypsy were scaled to the oldest insertion found at ~6.4 Mya, and a heatmap corresponding to the age of insertion along the chromosomes, <0.6 Mya (red) and >0.6 Mya (blue). Insertion times over chromosomes 2H and 7H were plotted with horizon plots in karyoploteR (Gel and Serra, 2017; Hyndman *et al.*, 2023; R Core Team, 2024).

Identifying telomeric ends and estimating centromeric positions

We identified telomeric sequences in the assembly by aligning the sequence TTTAGGGx8 to the genomes of *H. erectifolium* and barley with BLAST+ 2.15.0: "blastn -task blastn-short" (Camacho *et al.*, 2009). The alignments were merged and interval gaps smaller than joined 1000 bp with bedtools (v2.31.0) : "bedtools merge -i -d 1000" (Quinlan and Hall, 2010). For estimation of centromeric regions and midpoint, we extracted the protein-coding domains of intact CRM LTR retrotransposon sequences

from the EDTA annotation of the genomes of *H. erectifolium* and MorexV3 with TEsorter (Zhang *et al.*, 2022). The CRM sequences were then aligned to the three genomes with BLAST+ 2.15.0: "blastn -task blastn -dust no -evalue 1e-50". Alignments were merged as before and the merged CRM segments were then divided into fragments of 100 bp with bedtools "bedtools makewindows -b $file -w 100" before importing and processing in R (v. 4.4.3) (Quinlan & Hall, 2010; R Core Team, 2024). The midpoint of the fragment density regions of CRM alignments was calculated with hdrcde (v. 3.4) in R and the 50 % confidence interval was chosen as a representative border of the centromere size and visualized with karyoploteR (Gel and Serra, 2017; Hyndman *et al.*, 2023).

Chromosomal synteny

Chromosomal synteny was calculated and visualized between *H. erectifolium* and barley, by mapping the genomes against one another with minimap2 (v. 2.28-r1209); "-K 5G -f 0.005 -eqx -c -x asm5" and Synteny and Rearrangement Identifier (SyRI) (v. 1.6.3) was used to calculate chromosomal alignments (Li, 2018; Goel *et al.*, 2019). We used plotsr (v. 1.1.0) "-s 1000000 -markers markers_cen_telo.txt -R -nodup -notr" to visualize and plot inversions >= 1 Mb, syntenic regions, telomeric ends, and centromeres (Goel & Schneeberger, 2022).

Quantification of PacBio IsoSeq data and tissue-specific expression with IsoQuant

We quantified transcript abundance of the 22 time-tissue specific PacBio IsoSeq samples with IsoQuant (v. 3.7.0) "--data_type pacbio_ccs --fl_data --fastq" to map and quantify the FLNC transcripts against the annotated *H. erectifolium* genome (Prjibelski *et al.*, 2023). We used the normalized transcripts per million (TPM) output of IsoQuant for Principal component analysis (PCA) in R with the built-in function prcomp in R (v. 4.4.3) and selected for visualization the top 20 genes contributing the most to principal component (PC) 1 (PC1) and PC2, and filtered for non-redundant genes (R Core Team, 2024).

Gene family evolution

A study of unique and shared genes and gene family evolution was made between *H. erectifolium* and nine additional species, including the two barley genotypes. Additional proteomes of seven species were retrieved from the JGI Phytozome database; *Arabidopsis thaliana* (L.) Heynh. (Athaliana_447_Araport11), *Sorghum bicolor* (L.) Moench (Sbicolor_730_v5.1), *Zea mays* L. (Zmays_833_Zm-B73-REFERENCE-NAM-5.0.55), *Oryza sativa* (Osativa_323_v7.0), *Brachypodium distachyon* (L.) P.Beauv. (Bdistachyon_556_v3.2), *Triticum aestivum* L. cv. Chinese Spring (Taestivumcv_ChineseSpring_725_v2.1), and *Thinopyrum intermedium* (Host) Barkworth & D.R.Dewey (Tintermedium_770_v3.1) (Goodstein *et al.*, 2012). The primary transcript of *H. erectifolium* as well as the longest transcript form MorexV3 were selected, and the "primaryTranscriptOnly" annotation files curated by JGI were used. The proteomes were assigned to hierarchical orthologous groups (HOG) with Orthofinder (v. 2.5.5): "-M msa -S blast" (Emms & Kelly,

2019). We visualized overlaps between species containing a minimum of 100 shared HOGs with ComplexUpset (v. 1.3.5) and made a functional enrichment analysis with Clusterprofiler of HOGs unique to *H. erectifolium* (v. 4.12.6) (Krassowski *et al.*, 2022; Xu *et al.*, 2024). For gene family evolution analysis, we used the species tree inferred by Orthofinder and selected the subtree without *A. thaliana* before converting it to an ultrametric tree, providing a separation time between *S. bicolor* and *H. erectifolium* of 59 Mya) "make_ultrametric.py -r 59000000", separation time was retrieved from TimeTree5 (Kumar *et al.*, 2022). The HOG gene counts at the root, N0, "orthogroup_gene_count.py N0.tsv", were calculated and HOGs gene counts differing by more than 60 genes between species were removed. The HOGs were then analyzed with CAFE5 (v. 1.1) with the additional parameters: "-p -k5" and when *Th. intermedium* was included parameter "-k3" was used (Mendes *et al.*, 2021). Finally, we used CafePlotter (v. 0.2.0): "-ignore_branch_length" to summarize and visualize the results from CAFE5 (moshi, 2024).

Cross-species drydown experiment

Experiments were performed in a controlled-environment Fitotron SGC Weiss Technik (Reiskirchen, Germany) growth chambers. Individual seeds were sown in 7×7×8 cm black plastic pots, 40 pots (5×8 rows) per tray, and each pot was filled with exactly 150 ±1 g of freshly prepared soil mixture. The plants of the two species were grown for either 8 or 2 weeks before the initiation of drydown to synchronise their initial biomass. The spring barley cultivar Morex was grown for two weeks under 12h photoperiod conditions (12h light, 12h dark, at 20°C day/16°C night, 60% relative humidity, photosynthetically active radiation ~300 µM m-2 s-1) before the start of the drydown. By contrast, *H. erectifolium* plants were grown for seven weeks at standard growth conditions. We then transferred the *H. erectifolium* plants for five days to 12h photoperiod conditions (12h light, 12h dark, at 20°C day/16°C night, 60% relative humidity, photosynthetically active radiation ~300 µM m-2 s-1) to synchronize the diurnal patterns of Morex and *H. erectifolium* plants before the start of drydown. Soil field capacity (FC) was gravimetrically calculated during each sowing. Fully hydrated soil, 100 % FC, was measured by having five prepared pots soak overnight and briefly let drain excess water before being weighed. They were then dried at 70 °C for 3 days, resulting in 0% FC. All pots were adjusted to 50 % FC before withholding water for six days for the drydown treatment, and control plants were maintained at 50 % FC daily. On the 6th day of treatment (DOT), water-withheld plants were re-watered to 50 % FC. Four replicate samples were collected from both control and drydown-treated plants at four timepoints, three during drydown on DOT 2, 5, and 6, the final sample was taken on DOT 7, 24 hours after re-watering. At each time point, four replicate samples were collected from the second leaf at ZT 8 for transcriptomic analyses, consisting of a pool of two plants per sample and frozen in liquid nitrogen. Plant fresh weight, soil FC, and leaf relative water content (RWC) was measured during each sampling. RWC was estimated as follows: sampled leaves were weighed, then soaked in dH_2O at 4 °C in the dark overnight for turgid weight and then dry weight after overnight drying at 70 °C. Calculated as: (fresh weight - dry

weight)/(turgid weight - dry weight = RWC) (Smart and Bingham, 1974). Total RNA was extracted with Qiagen RNeasy Plant Mini Kit (Qiagen, Hilden, Germany, Cat. No. / ID: 74904), with the addition of 0.2 % (v/v) beta-mercaptoethanol. RNA quantity and quality were assessed with NanoPhotometer NP80 (IMPLEN, Germany) and on a 1 % agarose gel. RNA samples were sequenced at Novogene UK (Cambridge, UK), Illumina PE150 sequenced with a minimum of 5 Gbp per sample.

Transcriptomic responses to drydown

The raw RNAseq sequencing data were quality controlled with FastQC (v. 0.12.1) and MulitQC (v. 1.12) to collate the reports (Andrews, 2010; Ewels *et al.*, 2016). We mapped the RNAseq reads with STAR (v. 2.7.11a) to their respective genomes "Genome index: STAR -runMode genomeGenerate -genomeFastaFiles *ASM* -sjdbGTFfile *GTF* -sjdbOverhang 149; RNAseq alignment: STAR -outSAMtype BAM SortedByCoordinate -quantMode TranscriptomeSAM GeneCounts" (Dobin *et al.*, 2013). Gene expression quantification was done with featureCounts (subread v. 2.0.6) "featureCounts -O -countReadPairs -p -G *ASM* -J -a *GTF*" (Liao *et al.*, 2014). The quantified read counts were normalized and filtered for lowly expressed genes using edgeR (v. 4.2.1) with the experimental setup and replicates as the group design (Chen *et al.*, 2025). A time-course analysis of transcriptomic changes in response to drydown and recovery over time was made with maSigPro (v. 1.76.0) using normalized reads from edgeR as input (Nueda *et al.*, 2014). We used the following criteria in maSigPro: "make.design.matrix(design, degree = 3); T.fit(family = negative.binomial(10), nvar.correction = TRUE) and filtered out a final set of genes with R^2 => 0.7. We used maSigPro to cluster the expression of genes found passing the R^2 threshold and identified coexpression clusters, and we manually assigned the time-course clusters into four groups based on their expression profiles. We then used edgeR to quantify transient DEGs at individual time-points, by comparing control-vs-drydown, in brief, glmQLFit(y, design, robust = TRUE: glmTreat(fit, contrast) for transient expression, $p \geq 0.01$. Functional enrichment analysis was made with Clusterprofiler (v. 4.12.6) and ComplexHeatmap (v. 2.20.0) for visualization of top biological processes functional enriched terms (Gu, 2022; Xu *et al.*, 2024). Single copy orthologs (SCO) between *H. erectifolium* and Morex were retrieved from HOGs, previous analysis with Orthofinder, and were further restricted by belonging to the same chromosome; these were used for direct DEG comparison between species.

Data analysis

Data wrangling, statistics, and visualization were performed in R (v. 4.4.3) (R Core Team, 2024), utilizing tidyverse (v 2.0.0), and statistics with agricole (v. 1.5) in (Wickham *et al.*, 2019; Mendiburu, 2023).

References

10x Genomics. 2024. *Long Ranger, version 2.2.2*. 10x Genomics. URL https://github.com/10XGenomics/longranger.

Andrews, S. 2010. *FastQC: a quality control tool for high throughout sequence data, version 0.12.1*. Babraham Bioinformatics. URL https://www.bioinformatics.babraham.ac.uk/projects/fastqc.

Aury J-M, Istace B. **2021**. Hapo-G, haplotype-aware polishing of genome assemblies with accurate reads. *NAR Genomics and Bioinformatics* **3**: lqab034.

Blum M, Chang H-Y, Chuguransky S, Grego T, Kandasaamy S, Mitchell A, Nuka G, Paysan-Lafosse T, Qureshi M, Raj S, *et al.* 2021. The InterPro protein families and domains database: 20 years on. *Nucleic Acids Research* **49**: D344–D354.

Bolger M, Schwacke R, Usadel B. **2021**. MapMan Visualization of RNA-Seq Data Using Mercator4 Functional Annotations. In: Dobnik D, Gruden K, Ramšak Ž, Coll A, eds. Solanum tuberosum: Methods and Protocols. New York, NY: Springer US, 195–212.

Buchfink B, Reuter K, Drost H-G. **2021**. Sensitive protein alignments at tree-of-life scale using DIAMOND. *Nature Methods* **18**: 366–368.

Camacho C, Coulouris G, Avagyan V, Ma N, Papadopoulos J, Bealer K, Madden TL. **2009**. BLAST+: architecture and applications. *BMC Bioinformatics* **10**: 1–9.

Camargo AP, Sourkov V, Pereira GAG, Carazzolle MF. **2020**. RNAsamba: neural network-based assessment of the protein-coding potential of RNA sequences. *NAR Genomics and Bioinformatics* **2**: lqz024.

Chen Y, Chen L, Lun ATL, Baldoni PL, Smyth GK. **2025**. edgeR v4: powerful differential analysis of sequencing data with expanded functionality and improved support for small counts and larger datasets. *Nucleic Acids Research* **53**: gkaf018.

Coplen TB, Brand WA, Gehre M, Gröning M, Meijer HAJ, Toman B, Verkouteren RM. **2006**. New Guidelines for δ13C Measurements. *Analytical Chemistry* **78**: 2439–2441.

Danecek P, Bonfield JK, Liddle J, Marshall J, Ohan V, Pollard MO, Whitwham A, Keane T, McCarthy SA, Davies RM, *et al.* 2021. Twelve years of SAMtools and BCFtools. *GigaScience* **10**: giab008.

De Coster W, Rademakers R. **2023**. NanoPack2: population-scale evaluation of long-read sequencing

data. *Bioinformatics* **39**: btad311.

Dobin A, Davis CA, Schlesinger F, Drenkow J, Zaleski C, Jha S, Batut P, Chaisson M, Gingeras TR. **2013**. STAR: ultrafast universal RNA-seq aligner. *Bioinformatics* **29**: 15–21.

Edith Schlagenhauf, Thomas Wicker. 2016. *The TREP platform: A curated database of transposable elements, version Release 2019*. URL https://trep-db.uzh.ch

Emms DM, Kelly S. **2019**. OrthoFinder: phylogenetic orthology inference for comparative genomics. *Genome Biology* **20**: 1–14.

Ewels P, Magnusson M, Lundin S, Käller M. **2016**. MultiQC: summarize analysis results for multiple tools and samples in a single report. *Bioinformatics* **32**: 3047–3048.

Fu L, Niu B, Zhu Z, Wu S, Li W. **2012**. CD-HIT: accelerated for clustering the next-generation sequencing data. *Bioinformatics* **28**: 3150–3152.

Gel B, Serra E. **2017**. karyoploteR: an R/Bioconductor package to plot customizable genomes displaying arbitrary data. *Bioinformatics* **33**: 3088–3090.

Goel M, Schneeberger K. **2022**. plotsr: visualizing structural similarities and rearrangements between multiple genomes. *Bioinformatics* **38**: 2922–2926.

Goel M, Sun H, Jiao W-B, Schneeberger K. **2019**. SyRI: finding genomic rearrangements and local sequence differences from whole-genome assemblies. *Genome Biology* **20**: 277.

Goodstein DM, Shu S, Howson R, Neupane R, Hayes RD, Fazo J, Mitros T, Dirks W, Hellsten U, Putnam N, *et al.* **2012**. Phytozome: a comparative platform for green plant genomics. *Nucleic Acids Research* **40**: D1178–D1186.

Gowik U, Bräutigam A, Weber KL, Weber APM, Westhoff P. **2011**. Evolution of C4 Photosynthesis in the Genus Flaveria: How Many and Which Genes Does It Take to Make C4? *The Plant Cell* **23**: 2087–2105.

Gu Z. **2022**. Complex heatmap visualization. *iMeta* **1**: e43.

Haas, B. 2023. *TransDecoder (Find Coding Regions Within Transcripts), versio*n *5.7.0.* URL https://github.com/TransDecoder/TransDecoder.

Himmelbach A, Ruban A, Walde I, Šimková H, Doležel J, Hastie A, Stein N, Mascher M. **2018a**. Discovery of multi-megabase polymorphic inversions by chromosome conformation capture

sequencing in large-genome plant species. *The Plant Journal* **96**: 1309–1316.

Himmelbach A, Walde I, Mascher M, Stein N. 2018b. Tethered Chromosome Conformation Capture Sequencing in Triticeae: A Valuable Tool for Genome Assembly. *Bio-protocol* **8**.

HMMER development team. 2020. *HMMER: Biosequence analysis using profile hidden Markov models, version 3.3.2*. URL http://hmmer.org.

Holst F, Bolger AM, Kindel F, Günther C, Maß J, Triesch S, Kiel N, Saadat N, Ebenhöh O, Usadel B, *et al.* 2025. Helixer: ab initio prediction of primary eukaryotic gene models combining deep learning and a hidden Markov model. *Nature Methods*: 1–8.

Hyndman, R. J., Einbeck, J., & Wand, M. P. 2023. *hdrcde: Highest Density Regions and Conditional Density Estimation, version 3.4*. URL https://pkg.robjhyndman.com/hdrcde.

Jackman SD, Coombe L, Chu J, Warren RL, Vandervalk BP, Yeo S, Xue Z, Mohamadi H, Bohlmann J, Jones SJM, *et al.* 2018. Tigmint: correcting assembly errors using linked reads from large molecules. *BMC Bioinformatics* **19**: 1–10.

Jacques Dainat, Darío Hereñú, Dr. K. D. Murray, Ed Davis, Ivan Ugrin, Kathryn Crouch, LucileSol, Nuno Agostinho, pascal-git, Zachary Zollman, & tayyrov. 2024. *Another Gtf/Gff Analysis Toolkit , version 1.2.0*. URL https://doi.org/10.5281/ZENODO.3552717.

Jayakodi M, Padmarasu S, Haberer G, Bonthala VS, Gundlach H, Monat C, Lux T, Kamal N, Lang D, Himmelbach A, *et al.* 2020. The barley pan-genome reveals the hidden legacy of mutation breeding. *Nature* **588**: 284–289.

Jin J, Lu P, Xu Y, Li Z, Yu S, Liu J, Wang H, Chua N-H, Cao P. **2021**. PLncDB V2.0: a comprehensive encyclopedia of plant long noncoding RNAs. *Nucleic Acids Research* **49**: D1489–D1495.

Jones P, Binns D, Chang H-Y, Fraser M, Li W, McAnulla C, McWilliam H, Maslen J, Mitchell A, Nuka G, *et al.* 2014. InterProScan 5: genome-scale protein function classification. *Bioinformatics* **30**: 1236–1240.

Kang Y-J, Yang D-C, Kong L, Hou M, Meng Y-Q, Wei L, Gao G. **2017**. CPC2: a fast and accurate coding potential calculator based on sequence intrinsic features. *Nucleic Acids Research* **45**: W12–W16.

Kolmogorov M, Yuan J, Lin Y, Pevzner PA. **2019**. Assembly of long, error-prone reads using repeat graphs. *Nature Biotechnology* **37**: 540–546.

Krassowski, M., Arts, M., Lagger, C., & Max. 2022. *ComplexUpset , version 1.3.5*. URL https://doi.org/10.5281/zenodo.7314197.

Kumar S, Suleski M, Craig JM, Kasprowicz AE, Sanderford M, Li M, Stecher G, Hedges SB. **2022**. TimeTree 5: An Expanded Resource for Species Divergence Times. *Molecular Biology and Evolution* **39**: msac174.

Li H. **2018**. Minimap2: pairwise alignment for nucleotide sequences. *Bioinformatics* **34**: 3094–3100.

Liao Y, Smyth GK, Shi W. **2014**. featureCounts: an efficient general purpose program for assigning sequence reads to genomic features. *Bioinformatics* **30**: 923–930.

Ma J, Bennetzen JL. **2004**. Rapid recent growth and divergence of rice nuclear genomes. *Proceedings of the National Academy of Sciences* **101**: 12404–12410.

Manni M, Berkeley MR, Seppey M, Simão FA, Zdobnov EM. **2021**. BUSCO Update: Novel and Streamlined Workflows along with Broader and Deeper Phylogenetic Coverage for Scoring of Eukaryotic, Prokaryotic, and Viral Genomes. *Molecular Biology and Evolution* **38**: 4647–4654.

Marçais G, Kingsford C. **2011**. A fast, lock-free approach for efficient parallel counting of occurrences of *k*-mers. *Bioinformatics* **27**: 764–770.

Mascher M, Wicker T, Jenkins J, Plott C, Lux T, Koh CS, Ens J, Gundlach H, Boston LB, Tulpová Z, *et al.* **2021**. Long-read sequence assembly: a technical evaluation in barley. *The Plant Cell* **33**: 1888–1906.

Mendes FK, Vanderpool D, Fulton B, Hahn MW. **2021**. CAFE 5 models variation in evolutionary rates among gene families. *Bioinformatics* **36**: 5516–5518.

Mendiburu, F. de. 2023. *agricolae: Statistical Procedures for Agricultural Research, version 1.5.0*. URL https://cran.r-project.org/web/packages/agricolae/index.html.

Mikheenko A, Prjibelski A, Saveliev V, Antipov D, Gurevich A. **2018**. Versatile genome assembly evaluation with QUAST-LG. *Bioinformatics* **34**: i142–i150.

Monat C, Padmarasu S, Lux T, Wicker T, Gundlach H, Himmelbach A, Ens J, Li C, Muehlbauer GJ, Schulman AH, *et al.* **2019**. TRITEX: chromosome-scale sequence assembly of Triticeae genomes with open-source tools. *Genome Biology* **20**: 1–18.

moshi. 2024. *CafePlotter , version 0.2.0*. URL https://github.com/moshi4/CafePlotter.

Negri T da C, Alves WAL, Bugatti PH, Saito PTM, Domingues DS, Paschoal AR. **2019**. Pattern recognition analysis on long noncoding RNAs: a tool for prediction in plants. *Briefings in Bioinformatics* **20**: 682–689.

Nueda MJ, Tarazona S, Conesa A. **2014**. Next maSigPro: updating maSigPro bioconductor package for RNA-seq time series. *Bioinformatics* **30**: 2598–2602.

Ou S, Jiang N. **2018**. LTR_retriever: A Highly Accurate and Sensitive Program for Identification of Long Terminal Repeat Retrotransposons. *Plant Physiology* **176**: 1410–1422.

Ou S, Su W, Liao Y, Chougule K, Agda JRA, Hellinga AJ, Lugo CSB, Elliott TA, Ware D, Peterson T, *et al.* 2019. Benchmarking transposable element annotation methods for creation of a streamlined, comprehensive pipeline. *Genome Biology* **20**: 1–18.

Oxford Nanopore Technologies. 2024. *Medaka, version 1.4.3*. Oxford Nanopore Technologies. URL https://github.com/nanoporetech/medaka

Oxford Nanopore Technologies. 2021. *Fever tree (Cinchona pubescens) leaf DNA*. Oxford Nanopore Technologies. [WWW document] URL https://nanoporetech.com/document/extraction-method/fever-tree-gdna. [accessed 10 September 2021].

Prjibelski AD, Mikheenko A, Joglekar A, Smetanin A, Jarroux J, Lapidus AL, Tilgner HU. **2023**. Accurate isoform discovery with IsoQuant using long reads. *Nature Biotechnology* **41**: 915–918.

Quinlan AR, Hall IM. **2010**. BEDTools: a flexible suite of utilities for comparing genomic features. *Bioinformatics* **26**: 841–842.

R Core Team. 2024. *R: A Language and Environment for Statistical Computing, version 4.4.3.* Vienna, Austria: R Foundation for Statistical Computing. URL https://www.R-project.org.

Rhie A, Walenz BP, Koren S, Phillippy AM. **2020**. Merqury: reference-free quality, completeness, and phasing assessment for genome assemblies. *Genome Biology* **21**: 1–27.

Šimková H, Tulpová Z, Cápal P. **2023**. Flow Sorting–Assisted Optical Mapping. In: Heitkam T, Garcia S, eds. *Plant Cytogenetics and Cytogenomics: Methods and Protocols*. New York, NY: Springer US, 465–483.

Singh U, Khemka N, Rajkumar MS, Garg R, Jain M. **2017**. PLncPRO for prediction of long non-coding RNAs (lncRNAs) in plants and its application for discovery of abiotic stress-responsive lncRNAs in rice and chickpea. *Nucleic Acids Research* **45**: e183.

Smart RE, Bingham GE. **1974**. Rapid Estimates of Relative Water Content. *Plant Physiology* **53**: 258–260.

Sun H, Ding J, Piednoël M, Schneeberger K. **2018**. findGSE: estimating genome size variation within human and Arabidopsis using *k*-mer frequencies. *Bioinformatics* **34**: 550–557.

Sun L, Luo H, Bu D, Zhao G, Yu K, Zhang C, Liu Y, Chen R, Zhao Y. **2013**. Utilizing sequence intrinsic composition to classify protein-coding and long non-coding transcripts. *Nucleic Acids Research* **41**: e166.

The UniProt Consortium, Bateman A, Martin M-J, Orchard S, Magrane M, Ahmad S, Alpi E, Bowler-Barnett EH, Britto R, Bye-A-Jee H, *et al.* **2023**. UniProt: the Universal Protein Knowledgebase in 2023. *Nucleic Acids Research* **51**: D523–D531.

Tong X, Liu S. **2019**. CPPred: coding potential prediction based on the global description of RNA sequence. *Nucleic Acids Research* **47**: e43.

The Tree of Life Programme. 2025. *ToLID - Tree of Life Identifiers*. [WWW document] URL https://id.tol.sanger.ac.uk. [accessed 2 February 2025].

Vaser R, Sović I, Nagarajan N, Šikić M. **2017**. Fast and accurate de novo genome assembly from long uncorrected reads. *Genome Research* **27**: 737–746.

Vasimuddin Md, Misra S, Li H, Aluru S. **2019**. Efficient Architecture-Aware Acceleration of BWA-MEM for Multicore Systems. In: 2019 IEEE International Parallel and Distributed Processing Symposium (IPDPS). 314–324.

Waddington SR, Cartwright PM, WALL PC. **1983**. A Quantitative Scale of Spike Initial and Pistil Development in Barley and Wheat. *Annals of Botany* **51**: 119–130.

Wang L, Park HJ, Dasari S, Wang S, Kocher J-P, Li W. **2013**. CPAT: Coding-Potential Assessment Tool using an alignment-free logistic regression model. *Nucleic Acids Research* **41**: e74.

Wick RR, Judd LM, Gorrie CL, Holt KE. **2017**. Completing bacterial genome assemblies with multiplex MinION sequencing. *Microbial Genomics* **3**.

Wickham H. **2016**. *ggplot2: Elegant Graphics for Data Analysis*. Springer-Verlag New York.

Wickham H, Averick M, Bryan J, Chang W, McGowan LD, François R, Grolemund G, Hayes A, Henry L, Hester J, *et al.* **2019**. Welcome to the Tidyverse. *Journal of Open Source Software* **4**: 1686.

Xu S, Hu E, Cai Y, Xie Z, Luo X, Zhan L, Tang W, Wang Q, Liu B, Wang R, *et al.* 2024. Using clusterProfiler to characterize multiomics data. *Nature Protocols* **19**: 3292–3320.

Yang C, Yang L, Zhou M, Xie H, Zhang C, Wang MD, Zhu H. **2018**. LncADeep: an ab initio lncRNA identification and functional annotation tool based on deep learning. *Bioinformatics* **34**: 3825–3834.

Zhang R-G, Li G-Y, Wang X-L, Dainat J, Wang Z-X, Ou S, Ma Y. **2022**. TEsorter: An accurate and fast method to classify LTR-retrotransposons in plant genomes. *Horticulture Research* **9**: uhac017.
